# OpenProt 2021: deeper functional annotation of the coding potential of eukaryotic genomes

**DOI:** 10.1093/nar/gkaa1036

**Published:** 2020-11-12

**Authors:** Marie A Brunet, Jean-François Lucier, Maxime Levesque, Sébastien Leblanc, Jean-Francois Jacques, Hassan R H Al-Saedi, Noé Guilloy, Frederic Grenier, Mariano Avino, Isabelle Fournier, Michel Salzet, Aïda Ouangraoua, Michelle S Scott, François-Michel Boisvert, Xavier Roucou

**Affiliations:** Department of Biochemistry and Functional Genomics, Université de Sherbrooke, 3201 Jean Mignault, Sherbrooke, QC J1E 4K8, Canada; PROTEO, Quebec Network for Research on Protein Function, Structure, and Engineering, Université Laval, Quebec City, QC G1V0A6, Canada; Center for Computational Science, Université de Sherbrooke, Sherbrooke, QC J1K 2R1, Canada; Biology Department, Université de Sherbrooke, Sherbrooke, QC J1K 2R1, Canada; Center for Computational Science, Université de Sherbrooke, Sherbrooke, QC J1K 2R1, Canada; Biology Department, Université de Sherbrooke, Sherbrooke, QC J1K 2R1, Canada; Department of Biochemistry and Functional Genomics, Université de Sherbrooke, 3201 Jean Mignault, Sherbrooke, QC J1E 4K8, Canada; PROTEO, Quebec Network for Research on Protein Function, Structure, and Engineering, Université Laval, Quebec City, QC G1V0A6, Canada; Department of Biochemistry and Functional Genomics, Université de Sherbrooke, 3201 Jean Mignault, Sherbrooke, QC J1E 4K8, Canada; PROTEO, Quebec Network for Research on Protein Function, Structure, and Engineering, Université Laval, Quebec City, QC G1V0A6, Canada; Department of Biochemistry and Functional Genomics, Université de Sherbrooke, 3201 Jean Mignault, Sherbrooke, QC J1E 4K8, Canada; Department of Biochemistry and Functional Genomics, Université de Sherbrooke, 3201 Jean Mignault, Sherbrooke, QC J1E 4K8, Canada; PROTEO, Quebec Network for Research on Protein Function, Structure, and Engineering, Université Laval, Quebec City, QC G1V0A6, Canada; Center for Computational Science, Université de Sherbrooke, Sherbrooke, QC J1K 2R1, Canada; Biology Department, Université de Sherbrooke, Sherbrooke, QC J1K 2R1, Canada; Department of Biochemistry and Functional Genomics, Université de Sherbrooke, 3201 Jean Mignault, Sherbrooke, QC J1E 4K8, Canada; INSERM U1192, Laboratoire Protéomique, Réponse Inflammatoire & Spectrométrie de Masse (PRISM), Université de Lille, F-59000 Lille, France; INSERM U1192, Laboratoire Protéomique, Réponse Inflammatoire & Spectrométrie de Masse (PRISM), Université de Lille, F-59000 Lille, France; Informatics Department, Université de Sherbrooke, Sherbrooke, QC J1K 2R1, Canada; Department of Biochemistry and Functional Genomics, Université de Sherbrooke, 3201 Jean Mignault, Sherbrooke, QC J1E 4K8, Canada; Department of Immunology and Cellular Biology, Université de Sherbrooke, Sherbrooke, QC J1E 4K8, Canada; Department of Biochemistry and Functional Genomics, Université de Sherbrooke, 3201 Jean Mignault, Sherbrooke, QC J1E 4K8, Canada; PROTEO, Quebec Network for Research on Protein Function, Structure, and Engineering, Université Laval, Quebec City, QC G1V0A6, Canada

## Abstract

OpenProt (www.openprot.org) is the first proteogenomic resource supporting a polycistronic annotation model for eukaryotic genomes. It provides a deeper annotation of open reading frames (ORFs) while mining experimental data for supporting evidence using cutting-edge algorithms. This update presents the major improvements since the initial release of OpenProt. All species support recent NCBI RefSeq and Ensembl annotations, with changes in annotations being reported in OpenProt. Using the 131 ribosome profiling datasets re-analysed by OpenProt to date, non-AUG initiation starts are reported alongside a confidence score of the initiating codon. From the 177 mass spectrometry datasets re-analysed by OpenProt to date, the unicity of the detected peptides is controlled at each implementation. Furthermore, to guide the users, detectability statistics and protein relationships (isoforms) are now reported for each protein. Finally, to foster access to deeper ORF annotation independently of one’s bioinformatics skills or computational resources, OpenProt now offers a data analysis platform. Users can submit their dataset for analysis and receive the results from the analysis by OpenProt. All data on OpenProt are freely available and downloadable for each species, the release-based format ensuring a continuous access to the data. Thus, OpenProt enables a more comprehensive annotation of eukaryotic genomes and fosters functional proteomic discoveries.

## INTRODUCTION

Genome annotations are the cornerstones of all research endeavours as they define the proteomic landscape. In the face of such a crucial role, they enforce data-driven and arbitrary criteria to reduce spurious annotations to a minimum (1–3). For example, annotations are limited to open reading frames (ORFs) longer than 100 codons, a single coding sequence per transcript and an ATG start codon, excepted for previously characterized examples. These criteria substantially shape and limit the exploration of the proteome (1,4–6) and an ever-increasing number of studies report the need for deeper ORF annotation to better explore and understand cellular mechanisms (1,7–9). With the development of ribosome profiling (10), a technique to detect translation events throughout the genome, inaccuracies from current annotations have been experimentally proven (8,11–15). Building on ribosome profiling data, several small ORF repositories have been published to foster the functional characterization of these previously overlooked ORFs (16–18). These repositories have greatly participated in the incorporation of small ORFs in genome annotations (1,16). Yet, because they rely sometimes exclusively on ribosome profiling data analyses, these repositories suffer from the technical biases inherent to the experiment. This may hinder the detection of overlapping ORFs or ORFs in low-abundance transcripts or in repetitive regions (1,10,19,20). Furthermore, the accuracy of the detection of isoforms may vary depending on the algorithm used to mine the data (21–23).

Using a different approach, OpenProt (24) first predicts all possible ORFs within the transcriptome retrieved from two annotations [NCBI RefSeq (25) and Ensembl (26)] and then gathers supporting evidence. The prediction pipeline does not enforce a maximal length threshold, although it does filter for a minimal length of 30 codons and an AUG initiating codon. The predicted encoded proteins are then categorized as follows: RefProt or reference proteins are known proteins annotated in NCBI RefSeq (25), Ensembl (26) and/or UniProt (27); novel isoforms are unannotated proteins with a significant sequence identity to a RefProt from the same gene; and AltProts are unannotated proteins with no significant identity to a RefProt from the same gene. Finally, to assert confidence of the predicted proteins, OpenProt retrieves evidence for each annotated protein. These are *in silico* evidence (conservation and prediction of functional domains) and experimental evidence (translation evidence from ribosome profiling data and expression evidence from mass spectrometry (MS) data). OpenProt thus offers a deep annotation of the genome of 10 species, identifying novel isoforms and novel proteins in an unbiased yet data-driven manner.

**Figure 1.**
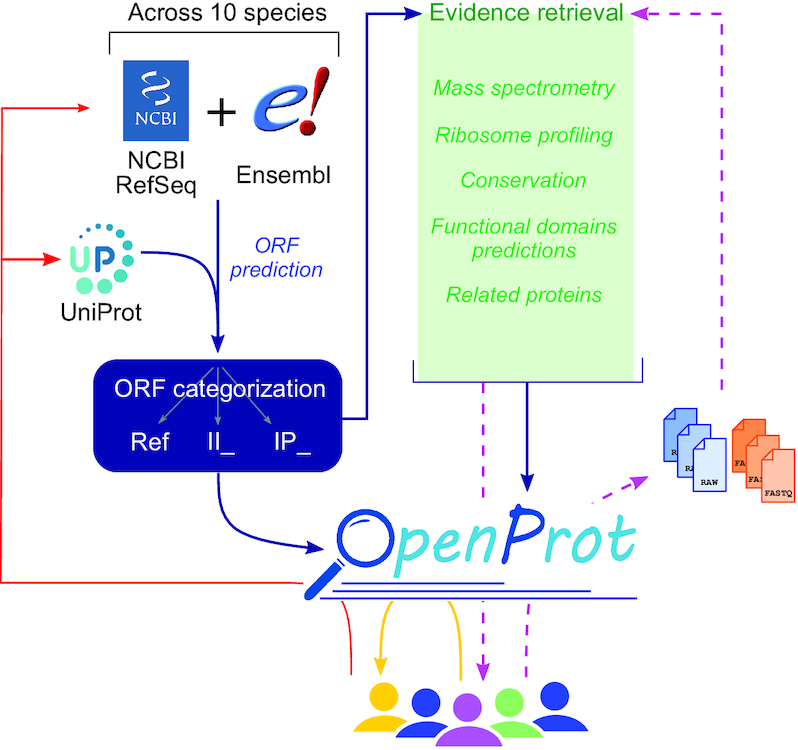
The OpenProt (v1.6) proteogenomic resource. OpenProt pipeline (dark blue arrows) contains two main features: prediction (on the left, blue) and evidence retrieval (middle, green). OpenProt enforces a polycistronic model of eukaryotic genes contrary to the actual dogma of one CDS per transcript. It retrieves all possible ORFs from transcripts annotated in NCBI RefSeq and/or Ensembl (ORF prediction, blue). The ORF-encoded proteins are then categorized as follows: RefProt if already annotated in NCBI RefSeq, Ensembl and/or UniProt; novel isoforms of known CDS (II_ accessions); or novel alternative proteins (IP_ accessions). All predicted proteins are available on the OpenProt website (www.openprot.org). Furthermore, OpenProt retrieves supporting evidence for all proteins from MS, ribosome profiling, protein conservation, functional domain prediction and related proteins. All data are inserted in the OpenProt resource and freely available to the community (yellow arrow). OpenProt also allows data submission by users for analysis using the OpenProt pipeline (purple dashed arrows). The results are then returned to the users and inserted in OpenProt at the next release (purple dashed arrows). With a symbiotic behaviour between the scientific community, linking experimental data, bioinformatics resources and deep ORF annotation, OpenProt participates in the implementation of novel, experimentally supported proteins in genome annotations and protein databases (red arrows).

The initial release of OpenProt provided a much needed resource for functional proteomic discoveries (7,28–32) and was the first proteogenomic resource supporting a polycistronic model of annotation for eukaryotic genomes (24). Here, we present an update of the OpenProt database incorporating the re-analysis of an additional 63 MS datasets and 44 ribosome profiling datasets, for a total of 177 and 131 datasets, respectively. This increase in the number of analysed datasets demonstrates the constant growth of the OpenProt resource. However, it does not constitute the core of this update. In this release, OpenProt implemented the transition to the latest annotations from NCBI RefSeq and Ensembl, the annotation of non-AUG initiation sites from ribosome profiling datasets, the incorporation of a time-independent quality control of the unicity of detected peptides and the annotation of protein identities within a gene (isoform prediction). Additionally, OpenProt website was upgraded to a more user-friendly interface and it now provides a platform for users to submit their datasets for re-analysis with the OpenProt pipeline. This platform aims to grant access to a deeper ORF annotation for data analysis to the wider community, independently of bioinformatics skills or computational resources. With a user-friendly and interactive interface, OpenProt (www.openprot.org) aims to foster discoveries of functional yet currently non-annotated proteins (Figure 1; Supplementary Material S1).

## DATABASE CONTENT UPDATES

### Genome annotations for ORF prediction

Genome annotations are constantly evolving, inserting novel transcripts, discarding others and updating annotations of coding sequence. Although most changes are minor from one release to the next, they do accumulate over time (3,33). The initial release of OpenProt was based on genome annotations from December 2015 for each species (24). These were updated to the latest release for each species as of January 2019. This major update necessitated an entire re-run of the OpenProt pipeline (Supplementary Material S1). For example, in humans alone 9873 messenger RNAs (mRNAs) and 2520 non-coding RNAs (ncRNAs) from the NCBI RefSeq annotation, and 1087 mRNAs and 371 ncRNAs from the Ensembl annotation were discarded or had their sequence changed. In parallel, 13 833 novel mRNAs and 2146 novel ncRNAs were included in the latest release of NCBI RefSeq, and 5970 novel mRNAs and 4884 novel ncRNAs in that of Ensembl. Consequently, 4256 RefProts from the NCBI RefSeq annotation and 2785 for Ensembl were discarded or had their amino acid sequence changed, while 4428 RefProts were added from NCBI RefSeq and 7182 from Ensembl. Genome assemblies, annotation releases and the associated number of predicted coding sequences for the OpenProt update are listed in Table 1.

**Table 1.** OpenProt (v1.6) prediction pipeline output

Species	Genome assembly	Annotations		ORFeome (both annotations)			
		NCBI RefSeq	Ensembl	Total #	Ref #	II_ #	IP_ #
*Homo sapiens*	GRCh38.p12	GRCh38.p12	GRCh38.95	692 045	134 477	68 612	488 956
*Pan troglodytes*	Pan_tro_3.0	Pan_tro_3.0	Pan_tro_3.0.95	331 247	79 070	14 308	237 869
*Mus musculus*	GRCm38.p6	GRCm38.p6	GRCm38.95	558 632	87 339	40 870	430 423
*Rattus norvegicus*	Rnor_6.0	Rnor_6.0	Rnor_6.0.95	294 727	51 662	7872	235 193
*Bos taurus*	ARS-UCD1.2	ARS-UCD1.2	ARS-UCD1.2.95	285 565	67 753	11 382	206 430
*Ovis aries*	Oar_v3.1	Oar_v3.1	Oar_v3.1.95	162 972	30 283	6339	126 350
*Danio rerio*	GRCz11	GRCz11	GRCz11.95	287 990	68 272	11 896	207 822
*Drosophila melanogaster*	Release 6 plus ISO1 MT	BDGP6	BDGP6.95	97 834	22 058	2125	73 651
*Caenorhabditis elegans*	WBcel235	WBcel235	WBcel235.95	94 890	28 516	3034	63 340
*Saccharomyces cerevisiae* S288c	R64-1-1	R64	R64-1-1.95	16 873	6615	28	10 230

Ref = currently annotated protein (RefProt); II_ = novel isoforms of known protein; IP_ = novel protein from alternative ORF (AltProt).

Interestingly, out of the newly included RefProts, 90 in NCBI RefSeq and 1835 in Ensembl had a length of ≤100 codons. These numbers reflect the efforts from the small ORF and alternative ORF community to foster annotation of these functional yet overlooked proteins (16,17,24). Thus, in humans 736 novel isoforms and 69 AltProts annotated in the OpenProt initial release are now RefProts since they have been included in annotations (NCBI RefSeq, Ensembl and/or UniProt). For example, the protein previously annotated IP_211724 in the initial release of OpenProt (https://openprot.org/p/savedSearch/jCa) was detected and described (renamed altDDIT3) in a publication building on OpenProt data (7). Consequently, the protein was included in the Ensembl annotation (ENSP00000494177) and in UniProt (A0A2R8YD15) in 2018, and became a RefProt in the latest OpenProt release (https://openprot.org/p/savedSearch/kCa). The numbers of AltProts and novel isoforms that have been included in NCBI RefSeq, Ensembl and/or UniProt since the initial release of OpenProt, across all supported species, are listed in Table 2.

**Table 2.** OpenProt fosters annotation of novel isoforms and novel proteins

Species	Predicted AltProt (OP v1.3), now RefProt (OP v1.6) (#)	Predicted novel isoform (OP v1.3), now RefProt (OP v1.6) (#)	Total changes (#)
*H. sapiens*	69	736	805
*P. troglodytes*	268	1702	1970
*M. musculus*	28	150	178
*R. norvegicus*	0	0	0
*B. taurus*	98	269	367
*O. aries*	0	0	0
*D. rerio*	155	661	816
*D. melanogaster*	1	1	2
*C. elegans*	8	51	59
*S. cerevisiae* S288c	0	0	0

OP = OpenProt; v1.3 = OpenProt version 1.3 [annotations from December 2015 (24)]; v1.6 = current OpenProt version (annotations from January 2019); RefProt = currently annotated protein; novel isoform = novel isoform of known protein; AltProt = novel protein from an alternative ORF.

### ORF calling from ribosome profiling datasets

In its initial release, OpenProt only reported evidence of translation for ORFs starting with an AUG. Yet pervasive translation with alternative start codons is increasingly reported by the community (14–17), with up to 83% of the sORF repository consisting of non-AUG small ORFs (28). The latest release of OpenProt now integrates data-driven annotation of non-AUG initiating codons. The pipeline of analysis for ribosome profiling datasets (Ribo-seq) uses the PRICE algorithm (21), which can accurately predict non-AUG start ORFs (Supplementary Material S1). Briefly, PRICE uses a logistic regression model to accurately predict initiating codons in Ribo-seq data, whether the dataset is enriched or not for initiating ribosomes. PRICE thus reconstitutes a set of codons most likely to yield the observed reads. We observed that when including all codons as possible initiation sites, the *P*-value of the reported ORF would increase as the overlap with the ORF predicted by OpenProt decreases (Supplementary Material S2). In PRICE, the *P*-value is the result of a generalized binomial test; thus, it indicates the confidence of that ORF not being attributable to noise. As the length of the ORF decreases, it is expected to be more difficult to distinguish true translation event from noise. In order to only annotate the most confident alternative initiation starts, we set the following filters based on our observation (Supplementary Material S2): any codon as initiating codon, and an overlap of the ORF candidate with the ORF predicted by OpenProt above 70%. Using these new filters, almost half a million (488 231) translation events could be retrieved in human datasets, corresponding to 33 836 ORFs across OpenProt.

### Peptide unicity in MS datasets

Unique and recurrent peptide detection in datasets of MS-based proteomics is considered a gold standard evidence of novel protein expression (1,34). However, the characterization of a unique peptide is often dependent on the database used at the time of the analysis. In classical MS analyses, a peptide is considered unique if it matches to one and only one protein. However, if a protein is not included in the database, it will not be taken into account in the evaluation of the unicity of the peptide. In the initial release of OpenProt, we ensured unicity by using an exhaustive proteome derived from both NCBI RefSeq and Ensembl annotations, as well as UniProt (24). Yet, as mentioned earlier, annotations evolve and with that the proteomic landscape. In the latest OpenProt release, we ensured a control of peptide unicity from MS datasets through time and releases. At each OpenProt release, the set of peptides mapped from all MS datasets to proteins is rescanned for protein assignation enforcing the OpenProt assignation rules. (i) When a peptide matches to multiple protein sequences from different genes, it is discarded. (ii) When a peptide matches to multiple protein sequences from the same gene, with at least one of the proteins being a RefProt, the peptide is given to the RefProt(s) only. (iii) When a peptide matches multiple AltProts or novel isoforms but not RefProts, the peptide is assigned to all AltProts and novel isoforms. Using such rules, OpenProt reports MS-based evidence of expression of novel proteins (AltProts and novel isoforms) only if the detected peptide does not match any known protein.

Furthermore, during this control of peptide unicity, the isobaric residues leucine and isoleucine are treated as undistinguishable. For example, the peptide EIGNLISDAMK was called by our SearchGUI/PeptideShaker MS pipeline to support the identification of IP_658154 (HSPD1P7). However, this peptide only differs by its leucine from the canonical protein of the parental gene HSPD1, which contains the peptide EIGNIISDAMK. Thus, this peptide was re-assigned as EIGNIISDAMK to the RefProt (P10809).

### MS statistics to guide data interpretation

Based on the OpenProt pipeline and stringent peptide assignation criteria regarding novel proteins, one must realize that not all predicted AltProts or novel isoforms are detectable by MS (35). To help the OpenProt users with the interpretation of an MS score of 0, we added MS coverage statistics to the MS tab of the details page for each protein. The coverage statistics tab contains the number of independent datasets in which the protein was detected, the number of unique peptides detected across all datasets and the number of peptide spectrum matches across all datasets. Alongside these metrics, the theoretical and current coverages by MS for each given protein are indicated (named possible sequence coverage and detected sequence coverage, respectively). The theoretical coverage is calculated from all possible tryptic peptides that would fit the OpenProt criteria to be assigned: a minimal length of seven amino acids, a maximal mass of 4600 Da and peptide unicity given the protein type (RefProt, novel isoform or AltProt). Thus, in *H. sapiens* 44 350 of OpenProt annotated proteins would not be detectable by MS, 13 595 in *P. troglodytes*, 31 275 in *M. musculus*, 11 311 in *R. norvegicus*, 11 454 in *B. taurus*, 8592 in *O. aries*, 15 294 in *D. rerio*, 1575 in *D. melanogaster*, 2267 in *C. elegans* and 245 in *S. cerevisiae* S288c.

A summary of experimental detections across all species supported by OpenProt is provided in Table 3 and general statistics are now provided in the About page of the OpenProt website (https://openprot.org/p/about; Supplementary Material S3) alongside a link to a stand-alone, freely accessible version of the script for calculating MS statistics.

**Table 3.** OpenProt (v1.6) evidence collection output

Species	Conservation evidence				Translation evidence (Ribo-seq)				Protein evidence (MS)			
	Sp #	Ref #	II_ #	IP_ #	St #	Ref #	II_ #	IP_ #	St #	Ref #	II_ #	IP_ #
*H. sapiens*	9	116 295	45 938	217 897	69	31 453	6912	9738	95	120 925	2446	40 883
*P. troglodytes*	9	73 137	13 315	131 321	0	N/A	N/A	N/A	2	25 821	15	360
*M. musculus*	9	78 193	25 867	98 047	40	23 740	3309	5908	40	66 875	236	4458
*R. norvegicus*	9	49 994	6012	77 768	3	16 205	996	1731	8	21 210	16	398
*B. taurus*	9	64 866	7148	83 993	0	N/A	N/A	N/A	4	20 120	6	80
*O. aries*	9	30 091	6223	73 034	0	N/A	N/A	N/A	4	8618	22	104
*D. rerio*	9	58 386	6826	26 669	5	11 186	490	299	8	32 286	27	220
*D. melanogaster*	9	13 567	757	477	3	5370	147	243	4	11 560	24	168
*C. elegans*	9	13 182	1064	450	5	12 796	396	196	6	13 332	38	96
*S. cerevisiae* S288c	9	2895	5	34	6	5429	4	269	6	4191	0	44

Sp = number of species evaluated for orthology relationships (not counting the queried species); St = number of studies re-analysed by OpenProt; Ref = currently annotated CDS (RefORF); II_ = novel isoforms of known CDS; IP_ = novel CDS from alternative ORF (AltORF); N/A = when no dataset has been re-analysed for this species yet (OpenProt release 1.2). Conservation evidence = all proteins with at least one ortholog in at least one species. Translation evidence = all ORFs detect in at least one detection by PRICE analysis of Ribo-seq data. Protein evidence = all proteins with at least one unique peptide in at least one study.

### Identification of similarities of coding sequences within genes

**Figure 2.**
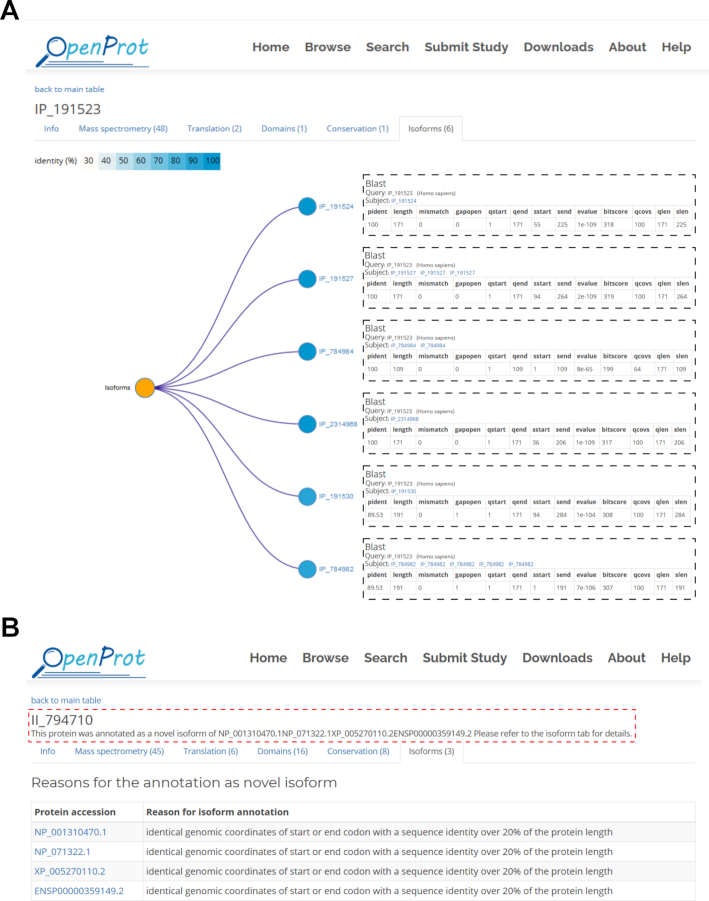
Annotation of novel protein relationships in OpenProt. (**A**) Content of the isoforms tab from the details page for IP_191523 on OpenProt (v1.6—available here). The protein accession always sits at the top left corner of the page. The isoform tab displayed here is the last tab of the page. The number indicated at the top corresponds to the number of identified isoforms. The tab contains a protein tree where nodes correspond to unique protein sequences present in OpenProt and are coloured based on their identity to the looked-up protein (here, IP_191523). Each node is accompanied by the corresponding protein accession. By clicking on the protein accession, a pop-up window (represented in dotted boxes) appears with the results of the BLAST search. (**B**) Content of the top of the isoforms tab from the details page for II_794710 on OpenProt (v1.6—available here). The protein accession always sits at the top left corner of the page. In the case of novel isoforms (accession starting with II_), a phrase under the accession indicates the RefProts responsible for the novel isoform annotation (circled in a red dotted line). The top of the isoform tab, displayed here, will thus contain an additional table. This table contains the accession of the RefProts responsible for the isoform annotation of the looked-up protein (here, II_794710), and the associated reason. Please note that because the filter for a novel protein to be annotated as novel isoform is more inclusive than that of the prediction of related proteins from the same gene, the RefProts listed in the table may not appear in the predicted isoform tree.

In this OpenProt update, the relationships between proteins from a same gene are now reported. In order to predict proteins as isoforms, OpenProt evaluates the protein sequence identity between proteins from a same gene using an all-versus-all BLAST (Basic Local Alignment Search Tool) (36). OpenProt reports proteins as isoforms if the result of their BLAST search yields a bit score over 40 for an overlap over 50% of the queried sequence, as previously published (37,38). The bit score was chosen as it is dependent on the size of the alignment, but not on the size of the database used. Thus, the higher the bit score, the better the sequence similarity. This isoform feature allows the user to grasp the complexity brought by alternative splicing and by the polycistronic nature of a transcript. For example, the AltProt IP_191523 from the *PIDD1* gene is related to six other AltProts from different transcripts of the *PIDD1* gene. These range from 284 amino acids (IP_191530) to 109 (IP_784984) (Figure 2A). In parallel, the canonical protein of the same transcript (ENSP00000416801) has eight isoforms ranging from 934 amino acids (XP_005253063) to 526 (Q9HB75-5).

Furthermore, OpenProt now also displays for novel isoforms (accessions starting with II_) the RefProt(s) responsible for the annotation as isoform instead of AltProt. This information is displayed at the top of the details page for each novel isoform and in detail in the isoforms tab. For example, the novel isoform II_794710 is annotated isoform because of four RefProts sharing identical genomic coordinates (start and/or end) and a sequence identity over 20% of the protein length. These are NP_001310470, NP_071322 and XP_005270110 from NCBI RefSeq, and ENSP00000359149 from Ensembl (Figure 2B).

### Database development

All data are generated using in-house Perl (version 5.18.2) and Python (version 3.6.9) scripts and stored in a PostgreSQL database (version 9.6). All re-analysed MS and ribosome profiling studies are accessible from the Help page (https://openprot.org/p/help; Supplementary Material S4).

## USER INTERFACE UPDATES

### Summary of the features presented in the initial release

At the time of the initial release, the OpenProt website provided three interfaces: a genome browser, a search page and a platform for downloads. The genome browser enables rapid lookup of all transcripts, proteins and detected peptides for a specific genomic region, while the search interface is designed for exploration of specific genes, transcripts and/or proteins. The platform for downloads allows custom retrieval of any data on the OpenProt resource as tsv, fasta or bed files (24,35). Each predicted ORF and encoded protein annotated in OpenProt has its own page that contains a genome browser centred on the dedicated ORF alongside all supporting information. All data from the initial release are still present on the website (under the release number 1.3); however, as genome annotations evolve, so does OpenProt, and we strongly encourage using the latest OpenProt release.

### Novel OpenProt web interface

The OpenProt resource is hosted at www.openprot.org since its initial release. The graphics of the website have, however, considerably changed for a more user-friendly and graphical interface. The home page (https://openprot.org/) now hosts a presentation of the project with tutorials and visual explanations. The query interface has been updated with an additional advanced search filter. The users can now filter results for specific MS or ribosome profiling datasets (Figure 1). Furthermore, a novel interface has been added for the submission of MS or ribosome profiling datasets to be analysed by OpenProt. This novel submission platform allows users to query their datasets for unannotated proteins, independently of their bioinformatics skills or computational resources. To ensure the quality of the data inserted into the OpenProt database, users first have to deposit their data into public repositories, the PRIDE archive (39) or ProteomeXchange (40) for MS datasets and Gene Omnibus (41) for ribosome profiling ones. After submission, the parameters are validated by the members of the OpenProt team prior to the analysis with the OpenProt pipeline (24) (Supplementary Material S1). Upon completion of the analysis, the results are sent to the users and the data are stored to be inserted in the next OpenProt release. For computational reasons, releases will be launched when 10 datasets are ready for implementation, or if it is 60 days since a dataset has been analysed. The Help page and contents have been updated to support this new interface (https://openprot.org/p/help; Supplementary Material S5).

### Website development

The OpenProt web platform was built using the Flask framework (version 1.1.2) and developed using HTML, SQL and JavaScript. The OpenProt website uses an HTTPS protocol to ensure protection of personal information. OpenProt is accessible via all major web browsers supporting JavaScript, such as Safari, Firefox, Chrome or Internet Explorer. All pages can be viewed on mobiles, but the interfaces have been optimized for display on computers or tablets.

## DISCUSSION AND COMPARISON TO EXISTING RESOURCES

The inaccuracies of current genome annotations have been increasingly reported in the last decades (1,4,14,28,29,42,43), and the OpenProt resource has offered a systematic approach to mend the gap between annotations and experimental data (24,35). OpenProt annotates thousands of novel predicted proteins supported by experimental evidence and functional predictions. As our pipeline evolves and more Ribo-seq and MS datasets are constantly added to the database, evidence for functional yet unannotated proteins is growing. At the time of this manuscript, the OpenProt resource lists 1477 novel proteins (AltProts or novel isoforms) in humans that were detected by both ribosome profiling and MS. Out of these, 935 are located on at least one mRNA that contains another annotated coding sequence. These numbers highlight not only the need for resources like the OpenProt database, but also the polycistronic nature of eukaryotic transcripts. Indeed, in each species for which OpenProt re-analysed at least one MS dataset and one ribosome profiling dataset, novel proteins detected by both methods and encoded in at least one mRNA containing another annotated sequence were reported. In particular, 98 were reported in *M. musculus*, 35 in *R. norvegicus*, 23 in *C. elegans*, 16 in *D. melanogaster*, 4 in *S. cerevisiae* and 2 in *D. rerio*. As the number of available datasets for these species increases, we expect these numbers of detection to rise.

To the best of our knowledge, OpenProt was the first database to fully endorse a polycistronic model of eukaryotic genome annotation. OpenProt differs from other small ORF databases (16,17) in that it does not uphold a maximum length threshold (below 100 codons for smORFs); it allows for multiple ORFs per transcript, supports two transcriptome annotations and allows for the identification and detection of novel isoforms of annotated proteins. Thus, OpenProt reaches a deeper ORF annotation throughout the genome. Furthermore, OpenProt now supports data submission to be analysed using the pipeline of OpenProt and with the results returned to the users. To the best of our knowledge, other small ORF databases do not offer this service although they may accept data suggestions (16).

In addition, OpenProt differentiates itself from UniProt (27) or NextProt (44) as it provides a genome browser and implements a polycistronic annotation model. Notwithstanding, as UniProt and NextProt are well-established proteomic resources, when a novel protein regroups sufficient evidence to meet UniProt and NextProt annotation requirements, it becomes a RefProt in the OpenProt database with the UniProt accession for ease of translation between databases. This symbiotic behaviour is exemplified by hundreds of novel proteins that have been annotated in NCBI RefSeq, Ensembl and/or UniProt since the first release of OpenProt (Table 2).

With the necessity of careful curation for the annotation of novel proteins comes the need for thorough and meticulous groundwork that annotation consortia are not designed to conduct (1,3,4). OpenProt and other small ORF resources are thus of upmost importance to foster less serendipitous discoveries of novel proteins and further our understanding of biological questions.

## FUTURE DIRECTIONS

Since its initial release (24), the OpenProt website counted over 36 000 visits and about 3000 downloads. The resource was used in 17 publications to discover novel proteins (5 of these were from our lab or collaborators) (30–32,45–57). This shows the OpenProt resource answered a need of the scientific community. The OpenProt development is heavily driven by suggestions from the community and recommendations of new features, species or experimental evidence are always welcome. These can be submitted via the OpenProt discussion forum.

The OpenProt pipeline is automated so that iGenomes updates and releases of NCBI RefSeq, Ensembl and/or UniProt are taken into account. The data are updated at the beginning of every odd year to ease management of computational resources access. OpenProt is a release-based platform, developed in accordance to the FAIR guiding principles for scientific data management and stewardship (58). This ensures an up-to-date, continuous availability of all OpenProt data through time. Furthermore, the shareable links created from the ‘Share’ feature at the top of the results table on the query interface are also release-based and persistent in time. This allows the inclusion of such links in publications for an exact snapshot of the OpenProt annotation at the time. All of the scripts behind OpenProt are available upon reasonable requests to the authors. Stand-alone scripts related to the mining of OpenProt data are available in the About page, under the Related Scripts section (https://openprot.org/p/about).

In future releases, OpenProt aims to tackle remaining challenges. For example, OpenProt is currently developing cutting-edge machine learning algorithms (e.g. convolutional neural networks) to more accurately predict coding sequences throughout genomes. Using such algorithms, OpenProt aims to insert ORFs shorter than 30 codons while avoiding spurious annotations. Furthermore, new tools and features will be added, such as a mass spectra viewer for evidence of protein detection, visualization of ribosomal coverage on the genome browser, networks of detected protein–protein interactions from affinity purification MS experiments and integration of genomic variants.

The quantity and quality of data provided by OpenProt along with its ease of use and transparent data availability hold potential to make it a long-lasting and popular proteogenomic resource.

## Supplementary Material

gkaa1036_Supplemental_File

## References

[1] Brunet M.A., Levesque S.A., Hunting D.J., Cohen A.A., Roucou X. Recognition of the polycistronic nature of human genes is critical to understanding the genotype–phenotype relationship. Genome Res. 2018; 28:609–624. 29626081 10.1101/gr.230938.117 PMC5932603

[2] Cheng H., Chan W.S., Li Z., Wang D., Liu S., Zhou Y. Small open reading frames: current prediction techniques and future prospect. Curr. Protein Pept. Sci. 2011; 12:503–507. 21787300 10.2174/138920311796957667 PMC3203329

[3] Mudge J.M., Harrow J. The state of play in higher eukaryote gene annotation. Nat. Rev. Genet. 2016; 17:758–772. 27773922 10.1038/nrg.2016.119 PMC5876476

[4] Orr M.W., Mao Y., Storz G., Qian S.-B. Alternative ORFs and small ORFs: shedding light on the dark proteome. Nucleic Acids Res. 2019; 48:1029–1042. 10.1093/nar/gkz734 PMC7026640 31504789

[5] Olexiouk V., Menschaert G. Identification of small novel coding sequences, a proteogenomics endeavor. Adv. Exp. Med. Biol. 2016; 926:49–64. 27686805 10.1007/978-3-319-42316-6_4

[6] Hellens R.P., Brown C.M., Chisnall M.A.W., Waterhouse P.M., Macknight R.C. The emerging world of small ORFs. Trends Plant Sci. 2016; 21:317–328. 26684391 10.1016/j.tplants.2015.11.005

[7] Samandi S., Roy A.V., Delcourt V., Lucier J.-F., Gagnon J., Beaudoin M.C., Vanderperre B., Breton M.-A., Motard J., Jacques J.-F. et al. Deep transcriptome annotation enables the discovery and functional characterization of cryptic small proteins. eLife. 2017; 6:e27860. 29083303 10.7554/eLife.27860 PMC5703645

[8] Menschaert G., Criekinge W.V., Notelaers T., Koch A., Crappé J., Gevaert K., Damme P.V. Deep proteome coverage based on ribosome profiling aids mass spectrometry-based protein and peptide discovery and provides evidence of alternative translation products and near-cognate translation initiation events. Mol. Cell. Proteomics. 2013; 12:1780–1790. 23429522 10.1074/mcp.M113.027540 PMC3708165

[9] Ma J., Ward C.C., Jungreis I., Slavoff S.A., Schwaid A.G., Neveu J., Budnik B.A., Kellis M., Saghatelian A. Discovery of human sORF-encoded polypeptides (SEPs) in cell lines and tissue. J. Proteome Res. 2014; 13:1757–1765. 24490786 10.1021/pr401280w PMC3993966

[10] Ingolia N.T. Ribosome profiling: new views of translation, from single codons to genome scale. Nat. Rev. Genet. 2014; 15:205–213. 24468696 10.1038/nrg3645

[11] Andreev D.E., O’Connor P.B.F., Zhdanov A.V., Dmitriev R.I., Shatsky I.N., Papkovsky D.B., Baranov P.V. Oxygen and glucose deprivation induces widespread alterations in mRNA translation within 20 minutes. Genome Biol. 2015; 16:90. 25943107 10.1186/s13059-015-0651-z PMC4419486

[12] Andreev D.E., O’Connor P.B.F., Fahey C., Kenny E.M., Terenin I.M., Dmitriev S.E., Cormican P., Morris D.W., Shatsky I.N., Baranov P.V. Translation of 5′ leaders is pervasive in genes resistant to eIF2 repression. eLife. 2015; 4:e03971. 25621764 10.7554/eLife.03971 PMC4383229

[13] Bazzini A.A., Johnstone T.G., Christiano R., Mackowiak S.D., Obermayer B., Fleming E.S., Vejnar C.E., Lee M.T., Rajewsky N., Walther T.C. et al. Identification of small ORFs in vertebrates using ribosome footprinting and evolutionary conservation. EMBO J. 2014; 33:981–993. 24705786 10.1002/embj.201488411 PMC4193932

[14] Chen J., Brunner A.-D., Cogan J.Z., Nuñez J.K., Fields A.P., Adamson B., Itzhak D.N., Li J.Y., Mann M., Leonetti M.D. et al. Pervasive functional translation of noncanonical human open reading frames. Science. 2020; 367:1140–1146. 32139545 10.1126/science.aay0262 PMC7289059

[15] Ingolia N.T. Ribosome footprint profiling of translation throughout the genome. Cell. 2016; 165:22. 27015305 10.1016/j.cell.2016.02.066 PMC4917602

[16] Olexiouk V., Van Criekinge W., Menschaert G. An update on sORFs.org: a repository of small ORFs identified by ribosome profiling. Nucleic Acids Res. 2018; 46:D497–D502. 29140531 10.1093/nar/gkx1130 PMC5753181

[17] Hao Y., Zhang L., Niu Y., Cai T., Luo J., He S., Zhang B., Zhang D., Qin Y., Yang F. et al. SmProt: a database of small proteins encoded by annotated coding and non-coding RNA loci. Brief. Bioinform. 2017; 19:636–643. 10.1093/bib/bbx005 28137767

[18] Xie S.-Q., Nie P., Wang Y., Wang H., Li H., Yang Z., Liu Y., Ren J., Xie Z. RPFdb: a database for genome wide information of translated mRNA generated from ribosome profiling. Nucleic Acids Res. 2016; 44:D254–D258. 26433228 10.1093/nar/gkv972 PMC4702944

[19] Ingolia N.T., Ghaemmaghami S., Newman J.R.S., Weissman J.S. Genome-wide analysis *in vivo* of translation with nucleotide resolution using ribosome profiling. Science. 2009; 324:218–223. 19213877 10.1126/science.1168978 PMC2746483

[20] Raj A., Wang S.H., Shim H., Harpak A., Li Y.I., Engelmann B., Stephens M., Gilad Y., Pritchard J.K. Thousands of novel translated open reading frames in humans inferred by ribosome footprint profiling. eLife. 2016; 5:e13328. 27232982 10.7554/eLife.13328 PMC4940163

[21] Erhard F., Halenius A., Zimmermann C., L’Hernault A., Kowalewski D.J., Weekes M.P., Stevanovic S., Zimmer R., Dölken L. Improved Ribo-seq enables identification of cryptic translation events. Nat. Methods. 2018; 15:363–366. 29529017 10.1038/nmeth.4631 PMC6152898

[22] Reixachs-Solé M., Ruiz-Orera J., Albà M.M., Eyras E. Ribosome profiling at isoform level reveals evolutionary conserved impacts of differential splicing on the proteome. Nat. Commun. 2020; 11:1768. 32286305 10.1038/s41467-020-15634-w PMC7156646

[23] Cui H., Hu H., Zeng J., Chen T. DeepShape: estimating isoform-level ribosome abundance and distribution with Ribo-seq data. BMC Bioinformatics. 2019; 20:678. 31861979 10.1186/s12859-019-3244-0 PMC6923924

[24] Brunet M.A., Brunelle M., Lucier J.-F., Delcourt V., Levesque M., Grenier F., Samandi S., Leblanc S., Aguilar J.-D., Dufour P. et al. OpenProt: a more comprehensive guide to explore eukaryotic coding potential and proteomes. Nucleic Acids Res. 2019; 47:D403–D410. 30299502 10.1093/nar/gky936 PMC6323990

[25] O’Leary N.A., Wright M.W., Brister J.R., Ciufo S., Haddad D., McVeigh R., Rajput B., Robbertse B., Smith-White B., Ako-Adjei D. et al. Reference sequence (RefSeq) database at NCBI: current status, taxonomic expansion, and functional annotation. Nucleic Acids Res. 2016; 44:D733–D745. 26553804 10.1093/nar/gkv1189 PMC4702849

[26] Yates A.D., Achuthan P., Akanni W., Allen J., Allen J., Alvarez-Jarreta J., Amode M.R., Armean I.M., Azov A.G., Bennett R. et al. Ensembl 2020. Nucleic Acids Res. 2020; 48:D682–D688. 31691826 10.1093/nar/gkz966 PMC7145704

[27] Consortium, T.U. UniProt: a worldwide hub of protein knowledge. Nucleic Acids Res. 2019; 47:D506–D515. 30395287 10.1093/nar/gky1049 PMC6323992

[28] Brunet M.A., Leblanc S., Roucou X. Reconsidering proteomic diversity with functional investigation of small ORFs and alternative ORFs. Exp. Cell Res. 2020; 393:112057. 32387289 10.1016/j.yexcr.2020.112057

[29] Peeters M., Menschaert G. The hunt for sORFs: a multidisciplinary strategy. Exp. Cell Res. 2020; 391:111923. 32135166 10.1016/j.yexcr.2020.111923

[30] Dubois M.-L., Meller A., Samandi S., Brunelle M., Frion J., Brunet M.A., Toupin A., Beaudoin M.C., Jacques J.-F., Lévesque D. et al. UBB pseudogene 4 encodes functional ubiquitin variants. Nat. Commun. 2020; 11:1306. 32161257 10.1038/s41467-020-15090-6 PMC7066184

[31] Cardon T., Franck J., Coyaud E., Laurent E.M.N., Damato M., Maffia M., Vergara D., Fournier I., Salzet M. Alternative proteins are functional regulators in cell reprogramming by PKA activation. Nucleic Acids Res. 2020; 48:7864–7882. 32324228 10.1093/nar/gkaa277 PMC7641301

[32] Cao X., Khitun A., Na Z., Dumitrescu D.G., Kubica M., Olatunji E., Slavoff S.A. Comparative proteomic profiling of unannotated microproteins and alternative proteins in human cell lines. J. Proteome Res. 2020; 19:3418–3426. 32449352 10.1021/acs.jproteome.0c00254 PMC7429271

[33] Salzberg S.L. Next-generation genome annotation: we still struggle to get it right. Genome Biol. 2019; 20:92. 31097009 10.1186/s13059-019-1715-2 PMC6521345

[34] Mann M. Origins of mass spectrometry-based proteomics. Nat. Rev. Mol. Cell Biol. 2016; 17:678. 10.1038/nrm.2016.135 27703242

[35] Brunet M.A., Lekehal A.M., Roucou X. How to illuminate the dark proteome using the multi-omic OpenProt resource. Curr. Protoc. Bioinformatics. 2020; 71:e103. 32780568 10.1002/cpbi.103

[36] Altschul S.F., Gish W., Miller W., Myers E.W., Lipman D.J. Basic local alignment search tool. J. Mol. Biol. 1990; 215:403–410. 2231712 10.1016/S0022-2836(05)80360-2

[37] Sonnhammer E.L.L., Östlund G. InParanoid 8: orthology analysis between 273 proteomes, mostly eukaryotic. Nucleic Acids Res. 2015; 43:D234–D239. 25429972 10.1093/nar/gku1203 PMC4383983

[38] Chen H., Shaw D., Zeng J., Bu D., Jiang T. DIFFUSE: predicting isoform functions from sequences and expression profiles via deep learning. Bioinformatics. 2019; 35:i284–i294. 31510699 10.1093/bioinformatics/btz367 PMC6612874

[39] Perez-Riverol Y., Csordas A., Bai J., Bernal-Llinares M., Hewapathirana S., Kundu D.J., Inuganti A., Griss J., Mayer G., Eisenacher M. et al. The PRIDE database and related tools and resources in 2019: improving support for quantification data. Nucleic Acids Res. 2019; 47:D442–D450. 30395289 10.1093/nar/gky1106 PMC6323896

[40] Deutsch E.W., Bandeira N., Sharma V., Perez-Riverol Y., Carver J.J., Kundu D.J., García-Seisdedos D., Jarnuczak A.F., Hewapathirana S., Pullman B.S. et al. The ProteomeXchange consortium in 2020: enabling ‘big data’ approaches in proteomics. Nucleic Acids Res. 2020; 48:D1145–D1152. 31686107 10.1093/nar/gkz984 PMC7145525

[41] Barrett T., Wilhite S.E., Ledoux P., Evangelista C., Kim I.F., Tomashevsky M., Marshall K.A., Phillippy K.H., Sherman P.M., Holko M. et al. NCBI GEO: archive for functional genomics data sets—update. Nucleic Acids Res. 2013; 41:D991–D995. 23193258 10.1093/nar/gks1193 PMC3531084

[42] Merino-Valverde I., Greco E., Abad M. The microproteome of cancer: from invisibility to relevance. Exp. Cell Res. 2020; 392:111997. 32302626 10.1016/j.yexcr.2020.111997

[43] Landry C.R., Zhong X., Nielly-Thibault L., Roucou X. Found in translation: functions and evolution of a recently discovered alternative proteome. Curr. Opin. Struct. Biol. 2015; 32:74–80. 25795211 10.1016/j.sbi.2015.02.017

[44] Zahn-Zabal M., Michel P.-A., Gateau A., Nikitin F., Schaeffer M., Audot E., Gaudet P., Duek P.D., Teixeira D., Rech de Laval V. et al. The neXtProt knowledgebase in 2020: data, tools and usability improvements. Nucleic Acids Res. 2020; 48:D328–D334. 31724716 10.1093/nar/gkz995 PMC7145669

[45] Cardon T., Salzet M., Franck J., Fournier I. Nuclei of HeLa cells interactomes unravel a network of ghost proteins involved in proteins translation. Biochim. Biophys. Acta: Gen. Subj. 2019; 1863:1458–1470. 31128158 10.1016/j.bbagen.2019.05.009

[46] Fesenko I., Kirov I., Kniazev A., Khazigaleeva R., Lazarev V., Kharlampieva D., Grafskaia E., Zgoda V., Butenko I., Arapidi G. et al. Distinct types of short open reading frames are translated in plant cells. Genome Res. 2019; 29:1464–1477. 31387879 10.1101/gr.253302.119 PMC6724668

[47] Brunet M.A., Roucou X. Mass spectrometry-based proteomics analyses using the OpenProt database to unveil novel proteins translated from non-canonical open reading frames. J. Vis. Exp. 2019; doi:10.3791/59589. 10.3791/59589 31033953

[48] Kiniry S.J., Michel A.M., Baranov P.V. Computational methods for ribosome profiling data analysis. WIREs RNA. 11:e1577. 10.1002/wrna.1577 31760685

[49] Cardon T., Hervé F., Delcourt V., Roucou X., Salzet M., Franck J., Fournier I. Optimized sample preparation workflow for improved identification of ghost proteins. Anal. Chem. 2020; 92:1122–1129. 31829555 10.1021/acs.analchem.9b04188

[50] Vergara D., Verri T., Damato M., Trerotola M., Simeone P., Franck J., Fournier I., Salzet M., Maffia M. A hidden human proteome signature characterizes the epithelial mesenchymal transition program. Curr. Pharm. Des. 2020; 26:372–375. 31995001 10.2174/1381612826666200129091610

[51] Wang B., Hao J., Pan N., Wang Z., Chen Y., Wan C. Identification and analysis of small proteins and short open reading frame encoded peptides in Hep3B cell. J. Proteomics. 2020; 230:103965. 32891891 10.1016/j.jprot.2020.103965

[52] Simoneau J., Gosselin R., Scott M.S. Factorial study of the RNA-seq computational workflow identifies biasesas technical gene signatures. NAR Genomics Bioinforma. 2020; 2:lqaa043. 10.1093/nargab/lqaa043 PMC7671328 33575596

[53] Erady C., Chong D., Meena N., Puntambekar S., Chauhan R., Umrania Y., Andreani A., Nel J., Wayland M.T., Pina C. et al. Translational products encoded by novel ORFs may form protein-like structures and have biological functions. 2019; bioRxiv doi:05 March 2019, preprint: not peer reviewed 10.1101/567800.

[54] Erady C., Puntambekar S., Prabakaran S. Use of short-read RNA-seq data to identify transcripts that can translate novel ORFs. 2020; bioRxiv doi:23 March 2020, preprint: not peer reviewed 10.1101/2020.03.21.001883.

[55] Brunet M.A., Jacques J.-F., Nassari S., Tyzack G.E., McGoldrick P., Zinman L., Jean S., Robertson J., Patani R., Roucou X. FUS gene is dual-coding with both proteins united in FUS-mediated toxicity. 2020; bioRxiv doi:14 April 2020, preprint: not peer reviewed 10.1101/848580. PMC7788448 33226175

[56] Jagannathan N.S., Meena N., Bhayankaram K.P., Prabakaran S. Proteins encoded by novel ORFs have increased disorder but can be biochemically regulated and harbour deleterious mutations. 2019; bioRxiv doi:29 March 2019, preprint: not peer reviewed 10.1101/562835.

[57] Murgoci A.-N., Cardon T., Aboulouard S., Duhamel M., Fournier I., Cizkova D., Salzet M. Reference and ghost proteins identification in rat C6 glioma extracellular vesicles. iScience. 2020; 23:101045. 32334413 10.1016/j.isci.2020.101045 PMC7182720

[58] Wilkinson M.D., Dumontier M., Aalbersberg I.J.J., Appleton G., Axton M., Baak A., Blomberg N., Boiten J.-W., da Silva Santos L.B., Bourne P.E. et al. The FAIR Guiding Principles for scientific data management and stewardship. Sci. Data. 2016; 3:160018. 26978244 10.1038/sdata.2016.18 PMC4792175

