# Supplementary material for: OpenProt 2021: deeper functional annotation of the coding potential of eukaryotic genomes
Source: Nucleic Acids Res. 2020 Nov 12;49(D1):D380–8. doi: 10.1093/nar/gkaa1036 (PMC7779043; doi:10.1093/nar/gkaa1036)
Supplement: gkaa1036_Supplemental_File [file gkaa1036_supplemental_file.pdf]

Supplementary Materials

*****

TABLE OF CONTENT *(click to get to the desired item)*

Supplementary S1: Summary of the methods behind all OpenProt features presented in the initial release.

Supplementary S2: Distribution of candidate ORF-pvalue based on the overlap with the ORF predicted by OpenProt.

Supplementary S3: General statistics per species for OpenProt v1.6.

Supplementary S4: List of mass spectrometry and ribosome profiling studies incorporated in OpenProt v1.6.

Supplementary S5: Getting started tutorials for OpenProt v1.6.

*****

Supplementary S1

Summary of the methods behind all OpenProt features presented in the initial release.

OpenProt is the first proteogenomic resource that supports a polycistronic annotation model of eukaryotic genomes. The database predicts all open reading frames (ORFs) from an exhaustive transcriptome, and categorizes all predicted ORFs as Reference proteins (RefProts - known proteins), Novel Isoforms (novel proteins with a sequence similarity with a known protein from the same gene) and Alternative proteins (AltProts - novel proteins with no sequence similarity with a known protein from the same gene). OpenProt then cumulates *in silico* and experimental evidence of expression for all predicted ORFs.

The OpenProt resource was first released in January 2019. A summary of the methods described in details in the initial release (24) is provided below. Similarly, tutorials for the web features (genome browser, search page, downloads and data submission platforms) are presented in **supplementary S5**.

Open Reading Frames (ORFs) prediction

OpenProt retrieves an exhaustive transcriptome by combining two well-used annotations (NCBI RefSeq and Ensembl), and predicts ORFs from a 3-frames in silico translation using EMBOSS Transeq. All possible ORFs with an ATG initiating codon and a minimal length of 30 codons are annotated and constitutes the OpenProt ORFeome. For each ORF, the source annotation (NCBI RefSeq or Ensembl) is associated to allow users to filter predictions by annotation if desired.

ORF product classification: RefProts, Novel Isoforms, and AltProts

The OpenProt ORFeome is then filtered using NCBI RefSeq, Ensembl and UniProt protein entries to identify annotated proteins, called RefProts. The identified RefProts are then associated the accession number from NCBI RefSeq, Ensembl and/or UniProt.
Once known ORFs are filtered out (RefProt category), only currently unannotated ORFs remain. All predicted proteins from the same gene of a RefProt are filtered as follows: (a) over 80 % of protein sequence identity with the RefProt over 50% of the length using Basic Local Alignment Search Tool (BLAST); (b) identical genomic coordinates of start or end codon with a sequence identity (EMBOSS Matcher PAM10 matrix score ≤ 100) over 20% of the length of the RefProt. If one of these two condition is met, the novel predicted protein is categorized as a Novel Isoform. The accession number of Novel Isoforms start with II_.
The remaining predicted proteins correspond to AltProts: novel proteins with no sequence similarity with a RefProt from the same gene. The accession number of AltProts start with IP_.
All predictions for each species are present on the OpenProt website and can be downloaded, queried or visualized using the Genome Browser. Comprehensive tutorials for all three platforms is provided in **supplementary S5** and under the Help section of the OpenProt website.

Mass spectrometry data analysis pipeline

OpenProt re-analyzes mass spectrometry (MS) datasets to gather evidence of expression for all proteins annotated in the database. MS datasets are downloaded from ProteomeXchange and the PRIDE archive. All datasets are re-analyzed using the OpenProt protein FASTA containing all RefProts, AltProts and Isoforms. The MS pipeline uses PeptideShaker software configured to run 4 search engines on mgf MS files (X!Tandem, MS-GF+, Comet and OMSSA) via SearchGUI. The SearchGUI general parameters are set as described in the original studies. To account for the increase of the search space when using such a large database as OpenProt, the FDR is set at 0.001%. Furthermore, peptide assignation rules are enforced such that a predicted protein (AltProt or Isoform) can only be identified with a unique peptide. When a peptide matches a novel predicted protein and a RefProt, it is assigned to the RefProt only.
The identification results from MS analyses are implemented to the OpenProt database and can be downloaded or queried from the Genome Browser or the Search page. More information on the MS pipeline can be found on the OpenProt Help page.

Ribosome profiling data analysis pipeline

OpenProt re-analyzes ribosome profiling (Ribo-seq) datasets to gather evidence of translation for all ORFs annotated in the database. Ribo-seq datasets are downloaded from the Gene Omnibus platform. The raw data are re-analyzed using the PRICE workflow. PRICE is run with default parameters, except for the FDR set at 1 % (instead of 10%) using the rescue mode, and fed with both NCBI RefSeq and Ensembl annotations (run separately). Briefly, reads mapping to ribosomal RNAs are filtered out and remaining footprints are mapped onto the genome and transcriptome with up to three mismatches. Multi-mapped reads are fractionated across all possible sites unless uniquely mapped reads to nearby loci allow confident identification of the footprint coordinates. PRICE reconstitutes the set of codons most likely to yield the observed reads, creating a list of ORF candidates. These are filtered according to a stringent 1% FDR (usually set at 10%) to focus on highly confident translation events.
The identification results from Ribo-seq analyses are implemented to the OpenProt database and can be downloaded or queried from the Search page. More information on the pipeline can be found on the OpenProt Help page.

Conservation analysis pipeline

OpenProt gathers evidence of conservation for all proteins annotated in the database. OpenProt computes orthology relationships from the 10 currently supported species. The homology of protein sequences is evaluated using an InParanoid-like approach and separates orthologs (homologous sequences from different species) from paralogs (homologous sequences from the same species but different genes). Orthologs are identified using an all-vs-all BLAST, i.e. all protein sequences from one species are searched against all protein sequences from another species. Similarly, all protein sequences from one species are searched against all protein sequences from a different gene of the same species to identify paralogs. Orthology relationships are called for a bit-score over 40 and an overlap over 50 % of the queried sequence.
All identified orthology relationships are then implemented to the OpenProt database and can be downloaded or queried from the Search page. More information can be found on the OpenProt Help page.

Protein functional domain prediction

All proteins annotated on OpenProt are scanned for the presence of known functional domains using InterProScan with the default parameters. Domain predictions as well as gene ontology (GO) and pathway annotations are reported if significant (e-value < 10-3).
The results are inserted into the OpenProt database and can be downloaded or queried from the Search page. More information are available on the OpenProt Help page.

Applications and Downloads

In addition to a genome Browser and an advanced query page, OpenProt website contains a Downloads platform. This platform allows customable downloads of all data present on OpenProt for every release.
For example, one can select only the most confident protein predictions (previously unannotated proteins detected with at least two unique peptides in mass spectrometry experiments), or all predicted proteins. Personalized database generation and download based on custom RNA-seq results is supported, as detailed in the Help page and a stand-alone script is available in the Related Scripts section of the About page.
The results table from specific queries made on the search page can be shared or downloaded as a TSV or a protein FASTA file (detailed in the Help page).

Overall, OpenProt allows (1) advanced search and download of results table, (2) genome browsing with visualization of MS evidence, and (3) personalized downloads (data and file format) for any research endeavour.

Back to the table of contents

<u>**Supplementary S2**</u>

Distribution of candidate ORF-pvalue based on the overlap with the ORF predicted by OpenProt

The PRICE algorithm associates a p-value to each ORF candidate. This p-value is the result of a generalized binomial test and as such relates to the confidence of the given ORF to not be attributable to noise. This was evaluated through all studies on OpenProt, but only the study with the top (lowest p-values, panel A) and bottom (highest p-values, panel B) results are presented.

A. From the study GSE64962 in human fibroblasts, the distribution of p-values of the ORF candidates from the PRICE algorithm are observed based on their minimal overlap with the corresponding ORF predicted by OpenProt. All codons are considered possible initiation sites here. The boxplot corresponds to the median and 5-95 % confidence interval. Please not the graph y axis is from 10^{-24} to 10^{0}.

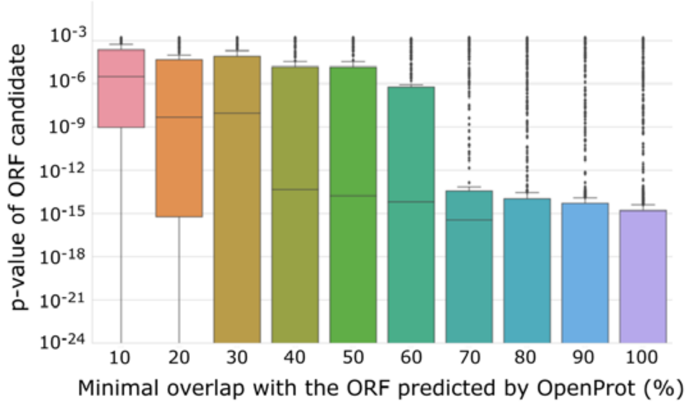

B. From the study GSE41605 in human fibroblasts, the distribution of p-values of the ORF candidates from the PRICE algorithm are observed based on their minimal overlap with the corresponding ORF predicted by OpenProt. All codons are considered possible initiation sites here. The boxplot corresponds to the median and 5-95 % confidence interval. Please not the graph y axis is from 10^{-24} to 10^{0}.

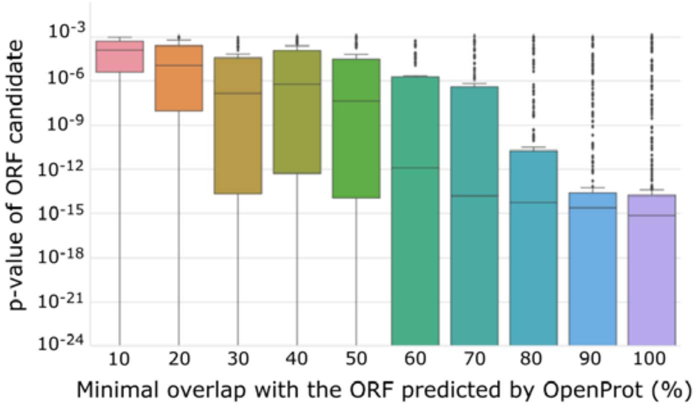

Back to the table of contents

Supplementary S3

General statistics per species for OpenProt v1.6

Here are the summary statistics for all species supported in OpenProt v1.6: *Homo sapiens*, *Pan troglodytes*, *Mus musculus*, *Rattus norvegicus*, *Bos taurus*, *Ovis aries*, *Danio rerio*, *Drosophila melanogaster*, *Caenorhabditis elegans* and *Saccharomyces cerevisiae*.

Legend for the pie charts: ncRNA = non-coding RNA; UTR = untranslated region of an mRNA; MS = mass spectrometry; TE = translation evidence from Ribo-seq.

*** OpenProt v1.6 general statistics in ***Homo sapiens*** ***

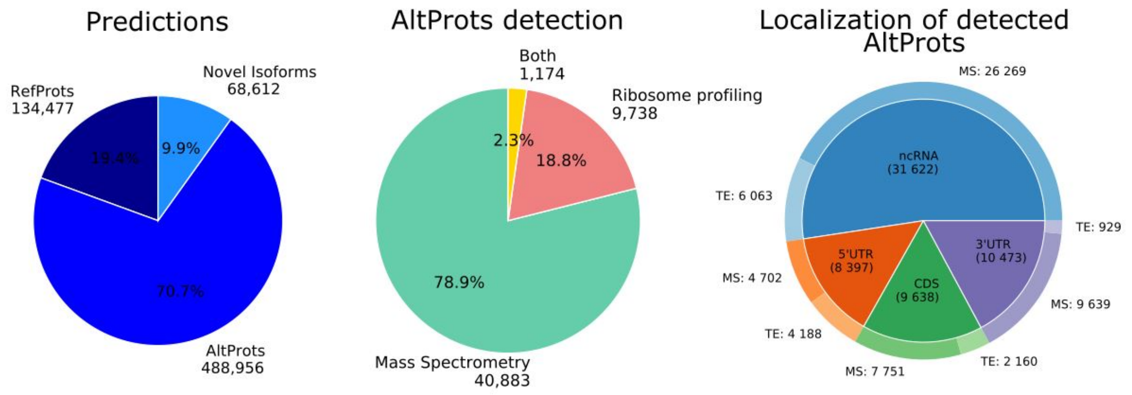

*** OpenProt v1.6 general statistics in ***Pan troglodytes*** ***

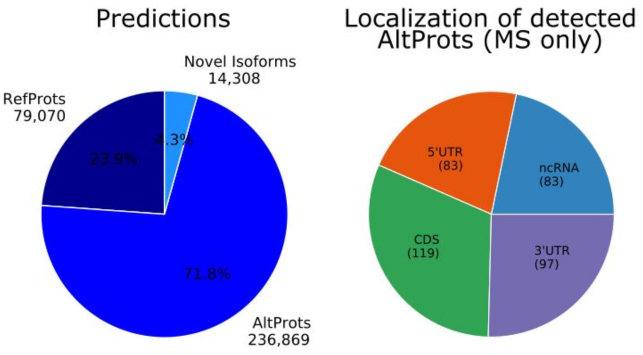

Please note that no ribosome profiling data was available for *Pan troglodytes*.

*** OpenProt v1.6 general statistics in ***Mus musculus*** ***

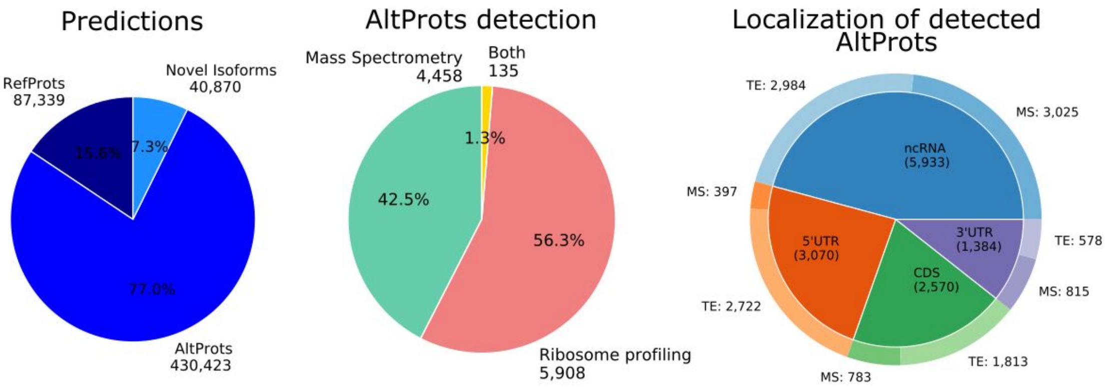

*** OpenProt v1.6 general statistics in ***Rattus norvegicus*** ***

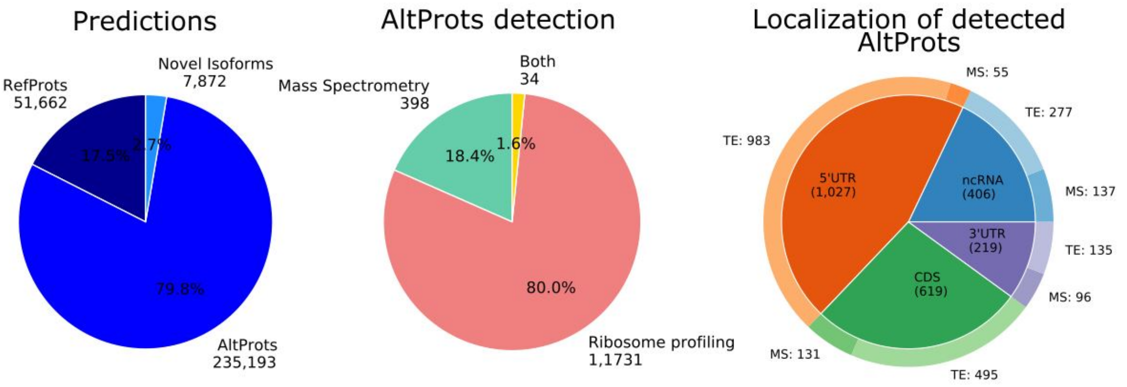

*** OpenProt v1.6 general statistics in ***Bos taurus*** ***

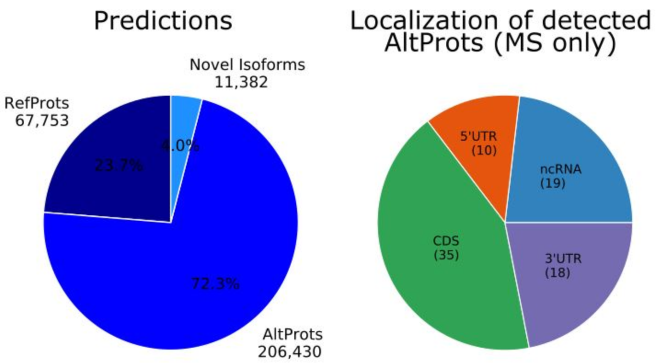

Please note that no ribosome profiling data was available for *Bos taurus*.

*** OpenProt v1.6 general statistics in ***Ovis aries***

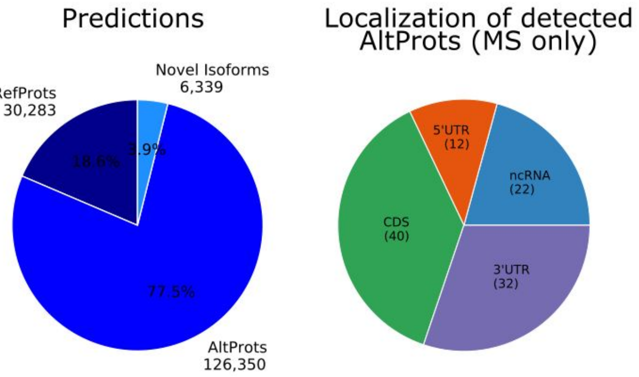

Please note that no ribosome profiling data was available for *Ovis aries*.

*** OpenProt v1.6 general statistics in ***Danio rerio***

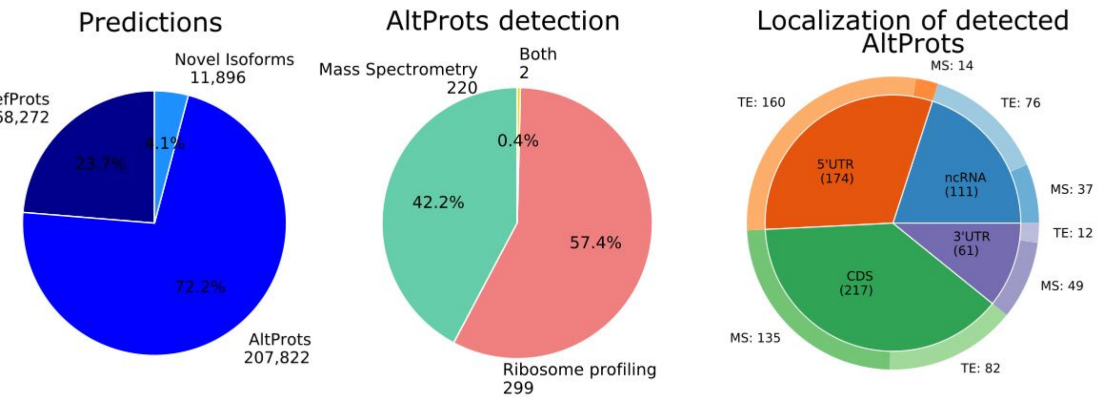

*** OpenProt v1.6 general statistics in ***Drosophila melanogaster*** ***

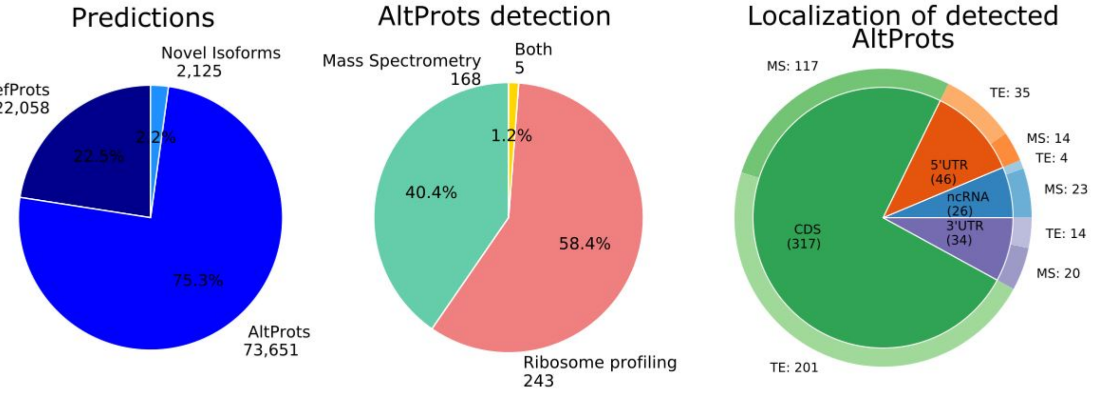

*** OpenProt v1.6 general statistics in ***Caenorhabditis elegans*** ***

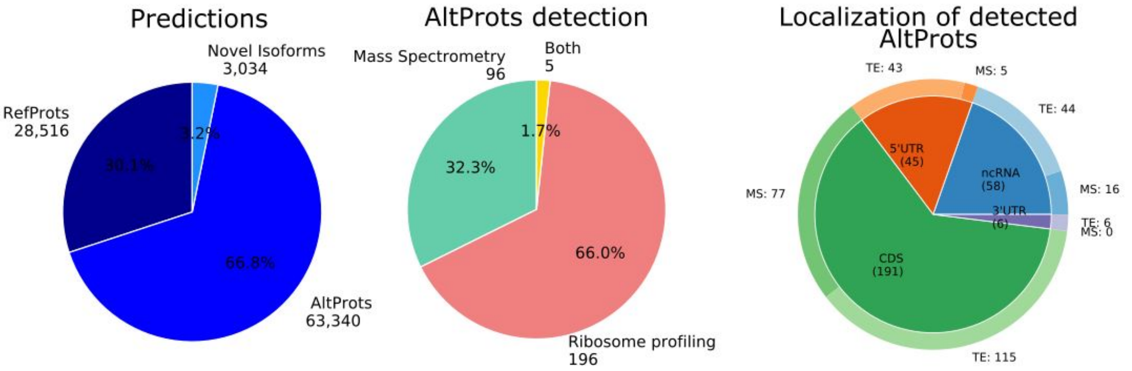

*** OpenProt v1.6 general statistics in ***Saccharomyces cerevisiae S288c*** ***

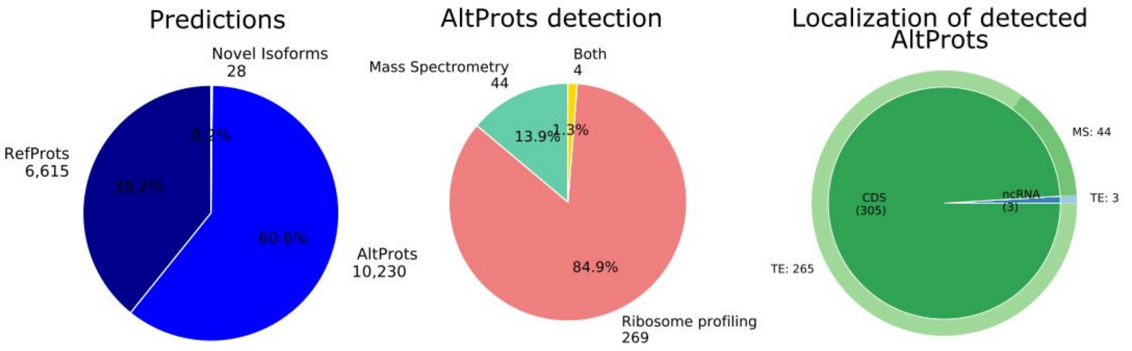

Back to the table of contents

Supplementary S4

List of mass spectrometry and ribosome profiling studies incorporated in OpenProt v1.6

List of the 177 **mass spectrometry** datasets incorporated in OpenProt v1.6 with the associated species and citation (PMID accession).

Species	Study ID	Data source	PMID
Saccharomyces cerevisiae	PXD000375	https://www.ebi.ac.uk/pride/archive/projects/PXD000375	24319056
Saccharomyces cerevisiae	PXD003736	https://www.ebi.ac.uk/pride/archive/projects/PXD003736	27251291
Saccharomyces cerevisiae	PXD003854	https://www.ebi.ac.uk/pride/archive/projects/PXD003854	27717283
Saccharomyces cerevisiae	PXD005795	https://www.ebi.ac.uk/pride/archive/projects/PXD005795	25767917
Saccharomyces cerevisiae	PXD006009	https://www.ebi.ac.uk/pride/archive/projects/PXD006009	28388408
Saccharomyces cerevisiae	PXD011929	https://www.ebi.ac.uk/pride/archive/projects/PXD011929	32284610
Homo sapiens	BioPlex_1	https://bioplex.hms.harvard.edu/	26186194
Homo sapiens	BioPlex_2	https://bioplex.hms.harvard.edu/	28514442
Homo sapiens	CCLE_2020	https://portals.broadinstitute.org/ccle/about	31978347
Homo sapiens	Chorus1043	https://chorusproject.org	27499296
Homo sapiens	CPTAC3_S041	https://cptac-data-portal.georgetown.edu/cptac/s/S041	29718670
Homo sapiens	CPTAC3_S044	https://cptac-data-portal.georgetown.edu/cptac/s/S044	31675502
Homo sapiens	CPTAC3_S051	https://cptac-data-portal.georgetown.edu/cptac/s/S051	31988290
Homo sapiens	CPTAC3_S053	https://cptac-data-portal.georgetown.edu/cptac/s/S053	32059776
Homo sapiens	MAC-tag	www.peptideatlas.org/PASS/PASS01076	29568061
Homo sapiens	MSV000082644	https://massive.ucsd.edu/ProteoSAFe/dataset.jsp?accession= MSV000082644	30205044
Homo sapiens	PRDB000035	https://www.proteomicsdb.org/#projects/35	23933261
Homo sapiens	PRDB004167	https://www.proteomicsdb.org/#projects/4167/3085	25892236
Homo sapiens	PXD000419	https://www.ebi.ac.uk/pride/archive/projects/PXD000419	24136357
Homo sapiens	PXD000529	https://www.ebi.ac.uk/pride/archive/projects/PXD000529	24328083
Homo sapiens	PXD000612	https://www.ebi.ac.uk/pride/archive/projects/PXD000612	25159151
Homo sapiens	PXD000788	https://www.ebi.ac.uk/pride/archive/projects/PXD000788	24797263
Homo sapiens	PXD001225	https://www.ebi.ac.uk/pride/archive/projects/PXD001225	28134274
Homo sapiens	PXD001406	https://www.ebi.ac.uk/pride/archive/projects/PXD001406	25657249
Homo sapiens	PXD001874	https://www.ebi.ac.uk/pride/archive/projects/PXD001874	26825538
Homo sapiens	PXD001889	https://www.ebi.ac.uk/pride/archive/projects/PXD001889	28248240
Homo sapiens	PXD001994	https://www.ebi.ac.uk/pride/archive/projects/PXD001994	26832662
Homo sapiens	PXD002214	https://www.ebi.ac.uk/pride/archive/projects/PXD002214	26371159
Homo sapiens	PXD002322	https://www.ebi.ac.uk/pride/archive/projects/PXD002322	26344197
Homo sapiens	PXD002516	https://www.ebi.ac.uk/pride/archive/projects/PXD002516	26892330
Homo sapiens	PXD002612	https://www.ebi.ac.uk/pride/archive/projects/PXD002612	27799870
Homo sapiens	PXD002815	https://www.ebi.ac.uk/pride/archive/projects/PXD002815	26496610
Homo sapiens	PXD002854	https://www.ebi.ac.uk/pride/archive/projects/PXD002854	27135364
Homo sapiens	PXD003115	https://www.ebi.ac.uk/pride/archive/projects/PXD003115	26657352
Homo sapiens	PXD003289	https://www.ebi.ac.uk/pride/archive/projects/PXD003289	28083997

Homo sapiens	PXD003431	https://www.ebi.ac.uk/pride/archive/projects/PXD003431	27852650
Homo sapiens	PXD003531	https://www.ebi.ac.uk/pride/archive/projects/PXD003531	28355574
Homo sapiens	PXD003668	https://www.ebi.ac.uk/pride/archive/projects/PXD003668	27561551
Homo sapiens	PXD003702	https://www.ebi.ac.uk/pride/archive/projects/PXD003702	27779199
Homo sapiens	PXD003708	https://www.ebi.ac.uk/pride/archive/projects/PXD003708	27987026
Homo sapiens	PXD003811	https://www.ebi.ac.uk/pride/archive/projects/PXD003811	28062795
Homo sapiens	PXD003902	https://www.ebi.ac.uk/pride/archive/projects/PXD003902	28007913
Homo sapiens	PXD003903	https://www.ebi.ac.uk/pride/archive/projects/PXD003903	28489815
Homo sapiens	PXD003937	https://www.ebi.ac.uk/pride/archive/projects/PXD003937	27790916
Homo sapiens	PXD003940	https://www.ebi.ac.uk/pride/archive/projects/PXD003940	27976581
Homo sapiens	PXD003947	https://www.ebi.ac.uk/pride/archive/projects/PXD003947	27444420
Homo sapiens	PXD003963	https://www.ebi.ac.uk/pride/archive/projects/PXD003963	28208246
Homo sapiens	PXD003965	https://www.ebi.ac.uk/pride/archive/projects/PXD003965	28208246
Homo sapiens	PXD003966	https://www.ebi.ac.uk/pride/archive/projects/PXD003966	28208246
Homo sapiens	PXD003967	https://www.ebi.ac.uk/pride/archive/projects/PXD003967	28208246
Homo sapiens	PXD003971	https://www.ebi.ac.uk/pride/archive/projects/PXD003971	28208246
Homo sapiens	PXD004242	https://www.ebi.ac.uk/pride/archive/projects/PXD004242	28007936
Homo sapiens	PXD004352	https://www.ebi.ac.uk/pride/archive/projects/PXD004352	28263321
Homo sapiens	PXD004424	https://www.ebi.ac.uk/pride/archive/projects/PXD004424	27723984
Homo sapiens	PXD004437	https://www.ebi.ac.uk/pride/archive/projects/PXD004437	27890679
Homo sapiens	PXD004452	https://www.ebi.ac.uk/pride/archive/projects/PXD004452	28601559
Homo sapiens	PXD004541	https://www.ebi.ac.uk/pride/archive/projects/PXD004541	27827301
Homo sapiens	PXD004624	https://www.ebi.ac.uk/pride/archive/projects/PXD004624	27879288
Homo sapiens	PXD004625	https://www.ebi.ac.uk/pride/archive/projects/PXD004625	27879288
Homo sapiens	PXD004626	https://www.ebi.ac.uk/pride/archive/projects/PXD004626	27879288
Homo sapiens	PXD004655	https://www.ebi.ac.uk/pride/archive/projects/PXD004655	28117667
Homo sapiens	PXD004682	https://www.ebi.ac.uk/pride/archive/projects/PXD004682	28195392
Homo sapiens	PXD004683	https://www.ebi.ac.uk/pride/archive/projects/PXD004683	28195392
Homo sapiens	PXD004710	https://www.ebi.ac.uk/pride/archive/projects/PXD004710	27801565
Homo sapiens	PXD004724	https://www.ebi.ac.uk/pride/archive/projects/PXD004724	28242843
Homo sapiens	PXD004764	https://www.ebi.ac.uk/pride/archive/projects/PXD004764	28071820
Homo sapiens	PXD004778	https://www.ebi.ac.uk/pride/archive/projects/PXD004778	28071820
Homo sapiens	PXD004788	https://www.ebi.ac.uk/pride/archive/projects/PXD004788	28071820
Homo sapiens	PXD004796	https://www.ebi.ac.uk/pride/archive/projects/PXD004796	28071820
Homo sapiens	PXD004816	https://www.ebi.ac.uk/pride/archive/projects/PXD004816	28123004
Homo sapiens	PXD004859	https://www.ebi.ac.uk/pride/archive/projects/PXD004859	27976366
Homo sapiens	PXD004875	https://www.ebi.ac.uk/pride/archive/projects/PXD004875	27779380
Homo sapiens	PXD004900	https://www.ebi.ac.uk/pride/archive/projects/PXD004900	27806443
Homo sapiens	PXD005021	https://www.ebi.ac.uk/pride/archive/projects/PXD005021	28265047
Homo sapiens	PXD005080	https://www.ebi.ac.uk/pride/archive/projects/PXD005080	28348171
Homo sapiens	PXD005083	https://www.ebi.ac.uk/pride/archive/projects/PXD005083	28196878
Homo sapiens	PXD005123	https://www.ebi.ac.uk/pride/archive/projects/PXD005123	27892468
Homo sapiens	PXD005210	https://www.ebi.ac.uk/pride/archive/projects/PXD005210	27794609
Homo sapiens	PXD005276	https://www.ebi.ac.uk/pride/archive/projects/PXD005276	28237943
Homo sapiens	PXD005479	https://www.ebi.ac.uk/pride/archive/projects/PXD005479	27894104

Homo sapiens	PXD005814	https://www.ebi.ac.uk/pride/archive/projects/PXD005814	28258195
Homo sapiens	PXD005846	https://www.ebi.ac.uk/pride/archive/projects/PXD005846	28336715
Homo sapiens	PXD006537	https://www.ebi.ac.uk/pride/archive/projects/PXD006537	30137212
Homo sapiens	PXD010093	https://www.ebi.ac.uk/pride/archive/projects/PXD010093	30277781
Homo sapiens	PXD010138	https://www.ebi.ac.uk/pride/archive/projects/PXD010138	31207390
Homo sapiens	PXD010154	https://www.ebi.ac.uk/pride/archive/projects/PXD010154	30777892
Homo sapiens	PXD010756	https://www.ebi.ac.uk/pride/archive/projects/PXD010756	30833305
Homo sapiens	PXD011254	https://www.ebi.ac.uk/pride/archive/projects/PXD011254	30659192
Homo sapiens	PXD011797	https://www.ebi.ac.uk/pride/archive/projects/PXD011797	31308252
Homo sapiens	PXD011929	https://www.ebi.ac.uk/pride/archive/projects/PXD011929	32284610
Homo sapiens	PXD012101	https://www.ebi.ac.uk/pride/archive/projects/PXD012101	30768908
Homo sapiens	PXD014031	https://www.ebi.ac.uk/pride/archive/projects/PXD014031	32139545
Homo sapiens	PXD014083	https://www.ebi.ac.uk/pride/archive/projects/PXD014083	31592669
Homo sapiens	PXD014557	https://www.ebi.ac.uk/pride/archive/projects/PXD014557	31699905
Homo sapiens	PXD015850	https://www.ebi.ac.uk/pride/archive/projects/PXD015850	31614365
Homo sapiens	PXD017269	https://www.ebi.ac.uk/pride/archive/projects/PXD017269	32154592
Homo sapiens	Syn10142580	https://www.synapse.org/#!Synapse:syn10142580/wiki/449336	29191947
Homo sapiens	Syn10147608	https://www.synapse.org/#!Synapse:syn6126101/wiki/457943	28691493
Homo sapiens	TCGA_BRCA	https://cptac-data-portal.georgetown.edu/cptac/s/S029	27251275
Homo sapiens	TCGA_COCA	https://cptc-xfer.uis.georgetown.edu/publicData/Phase_II_Data/TCGA_Colorectal_Cancer_S_022/	25043054
Homo sapiens	TCGA_OVCA	https://cptac-data-portal.georgetown.edu/cptac/s/S026	27372738
Danio rerio	PXD002455	https://www.ebi.ac.uk/pride/archive/projects/PXD002455	26585961
Danio rerio	PXD003455	https://www.ebi.ac.uk/pride/archive/projects/PXD003455	27696471
Danio rerio	PXD004209	https://www.ebi.ac.uk/pride/archive/projects/PXD004209	27459904
Danio rerio	PXD004876	https://www.ebi.ac.uk/pride/archive/projects/PXD004876	27898262
Danio rerio	PXD005129	https://www.ebi.ac.uk/pride/archive/projects/PXD005129	29145436
Danio rerio	PXD005137	https://www.ebi.ac.uk/pride/archive/projects/PXD005137	29145436
Danio rerio	PXD006098	https://www.ebi.ac.uk/pride/archive/projects/PXD006098	28381614
Danio rerio	PXD011929	https://www.ebi.ac.uk/pride/archive/projects/PXD011929	32284610
Mus musculus	MSV000079361	ftp://massive.ucsd.edu/MSV000079361/	26638175
Mus musculus	PXD000288	https://www.ebi.ac.uk/pride/archive/projects/PXD000288	25616865
Mus musculus	PXD000501	https://www.ebi.ac.uk/pride/archive/projects/PXD000501	24753479
Mus musculus	PXD000666	https://www.ebi.ac.uk/pride/archive/projects/PXD000666	25193168
Mus musculus	PXD000747	https://www.ebi.ac.uk/pride/archive/projects/PXD000747	25205226
Mus musculus	PXD000867	https://www.ebi.ac.uk/pride/archive/projects/PXD000867	25470552
Mus musculus	PXD001007	https://www.ebi.ac.uk/pride/archive/projects/PXD001007	24946870
Mus musculus	PXD001250	https://www.ebi.ac.uk/pride/archive/projects/PXD001250	26523646
Mus musculus	PXD001293	https://www.ebi.ac.uk/pride/archive/projects/PXD001293	25504905
Mus musculus	PXD001404	https://www.ebi.ac.uk/pride/archive/projects/PXD001404	25338131
Mus musculus	PXD001514	https://www.ebi.ac.uk/pride/archive/projects/PXD001514	26139848
Mus musculus	PXD001597	https://www.ebi.ac.uk/pride/archive/projects/PXD001597	26227174
Mus musculus	PXD001641	https://www.ebi.ac.uk/pride/archive/projects/PXD001641	25643707
Mus musculus	PXD001729	https://www.ebi.ac.uk/pride/archive/projects/PXD001729	26239621
Mus musculus	PXD001792	https://www.ebi.ac.uk/pride/archive/projects/PXD001792	26280412

Mus musculus	PXD001859	https://www.ebi.ac.uk/pride/archive/projects/PXD001859	26258413
Mus musculus	PXD002152	https://www.ebi.ac.uk/pride/archive/projects/PXD002152	27302655
Mus musculus	PXD002156	https://www.ebi.ac.uk/pride/archive/projects/PXD002156	26080680
Mus musculus	PXD002466	https://www.ebi.ac.uk/pride/archive/projects/PXD002466	27625594
Mus musculus	PXD002582	https://www.ebi.ac.uk/pride/archive/projects/PXD002582	26229149
Mus musculus	PXD002896	https://www.ebi.ac.uk/pride/archive/projects/PXD002896	26732734
Mus musculus	PXD002925	https://www.ebi.ac.uk/pride/archive/projects/PXD002925	27027324
Mus musculus	PXD002927	https://www.ebi.ac.uk/pride/archive/projects/PXD002927	27180971
Mus musculus	PXD003155	https://www.ebi.ac.uk/pride/archive/projects/PXD003155	26850065
Mus musculus	PXD003442	https://www.ebi.ac.uk/pride/archive/projects/PXD003442	26900923
Mus musculus	PXD003555	https://www.ebi.ac.uk/pride/archive/projects/PXD003555	27490109
Mus musculus	PXD003656	https://www.ebi.ac.uk/pride/archive/projects/PXD003656	26969716
Mus musculus	PXD004072	https://www.ebi.ac.uk/pride/archive/projects/PXD004072	27188442
Mus musculus	PXD004087	https://www.ebi.ac.uk/pride/archive/projects/PXD004087	27629805
Mus musculus	PXD004152	https://www.ebi.ac.uk/pride/archive/projects/PXD004152	28328168
Mus musculus	PXD004612	ftp://ftp.pride.ebi.ac.uk/pride/data/archive/2017/01/PXD004612	28071813
Mus musculus	PXD005184	https://www.ebi.ac.uk/pride/archive/projects/PXD005184	27806573
Mus musculus	PXD005312	https://www.ebi.ac.uk/pride/archive/projects/PXD005312	28066266
Mus musculus	PXD005449	https://www.ebi.ac.uk/pride/archive/projects/PXD005449	28174279
Mus musculus	PXD005492	https://www.ebi.ac.uk/pride/archive/projects/PXD005492	28285833
Mus musculus	PXD005635	https://www.ebi.ac.uk/pride/archive/projects/PXD005635	28345880
Mus musculus	PXD006002	https://www.ebi.ac.uk/pride/archive/projects/PXD006002	28362576
Mus musculus	PXD007974	https://www.ebi.ac.uk/pride/archive/projects/PXD007974	31926610
Mus musculus	PXD011304	https://www.ebi.ac.uk/pride/archive/projects/PXD011304	32325033
Mus musculus	PXD011929	https://www.ebi.ac.uk/pride/archive/projects/PXD011929	32284610
Rattus norvegicus	PXD001585	https://www.ebi.ac.uk/pride/archive/projects/PXD001585	25799991
Rattus norvegicus	PXD001984	https://www.ebi.ac.uk/pride/archive/projects/PXD001984	26330543
Rattus norvegicus	PXD001986	https://www.ebi.ac.uk/pride/archive/projects/PXD001986	26330543
Rattus norvegicus	PXD002188	https://www.ebi.ac.uk/pride/archive/projects/PXD002188	27053602
Rattus norvegicus	PXD003375	https://www.ebi.ac.uk/pride/archive/projects/PXD003375	27250205
Rattus norvegicus	PXD003520	https://www.ebi.ac.uk/pride/archive/projects/PXD003520	27358910
Rattus norvegicus	PXD004247	https://www.ebi.ac.uk/pride/archive/projects/PXD004247	27250205
Rattus norvegicus	PXD004889	https://www.ebi.ac.uk/pride/archive/projects/PXD004889	27764671
Drosophila melanogaster	PXD001455	https://www.ebi.ac.uk/pride/archive/projects/PXD001455	25403936
Drosophila melanogaster	PXD003755	https://www.ebi.ac.uk/pride/archive/projects/PXD003755	26823104
Drosophila melanogaster	PXD003944	https://www.ebi.ac.uk/pride/archive/projects/PXD003944	27956707
Drosophila melanogaster	PXD011929	https://www.ebi.ac.uk/pride/archive/projects/PXD011929	32284610
Caenorhabditis elegans	PXD011851	https://www.ebi.ac.uk/pride/archive/projects/PXD011851	30796049
Caenorhabditis elegans	PXD011859	https://www.ebi.ac.uk/pride/archive/projects/PXD011859	30796049
Caenorhabditis elegans	PXD011929	https://www.ebi.ac.uk/pride/archive/projects/PXD011929	32284610
Caenorhabditis elegans	PXD013720	https://www.ebi.ac.uk/pride/archive/projects/PXD013720	31527589
Caenorhabditis elegans	PXD014469	https://www.ebi.ac.uk/pride/archive/projects/PXD014469	31835171

Caenorhabditis elegans	PXD014573	https://www.ebi.ac.uk/pride/archive/projects/PXD014573	https://doi.org/10.1038/s42255-019-0123-y
Bos taurus	PXD001741	https://www.ebi.ac.uk/pride/archive/projects/PXD001741	25818294
Bos taurus	PXD014374	https://www.ebi.ac.uk/pride/archive/projects/PXD014374	32054912
Bos taurus	PXD015735	https://www.ebi.ac.uk/pride/archive/projects/PXD015735	31887207
Bos taurus	PXD016098	https://www.ebi.ac.uk/pride/archive/projects/PXD016098	32028040
Pan troglodytes	PXD000419	https://www.ebi.ac.uk/pride/archive/projects/PXD000419	24136357
Pan troglodytes	PXD015850	https://www.ebi.ac.uk/pride/archive/projects/PXD015850	31614365
Ovis aries	PXD004556	https://www.ebi.ac.uk/pride/archive/projects/PXD004556	27784645
Ovis aries	PXD004989	https://www.ebi.ac.uk/pride/archive/projects/PXD004989	28615994
Ovis aries	PXD013822	https://www.ebi.ac.uk/pride/archive/projects/PXD013822	31136077
Ovis aries	PXD014050	https://www.ebi.ac.uk/pride/archive/projects/PXD014050	31882954

List of the 131 **ribosome profiling** datasets incorporated in OpenProt v1.6 along the associated species, data source and citation (PMID accession).

Species	Study ID	Data source	PMID
Saccharomyces cerevisiae	GSE34082	https://www.ncbi.nlm.nih.gov/geo/query/acc.cgi?acc=GSE34082	22194413
Saccharomyces cerevisiae	GSE82220	https://www.ncbi.nlm.nih.gov/geo/query/acc.cgi?acc=GSE82220	27638886
Saccharomyces cerevisiae	GSE67387	https://www.ncbi.nlm.nih.gov/geo/query/acc.cgi?acc=GSE67387	26052047
Saccharomyces cerevisiae	GSE75322	https://www.ncbi.nlm.nih.gov/geo/query/acc.cgi?acc=GSE75322	26887592
Saccharomyces cerevisiae	GSE63789	https://www.ncbi.nlm.nih.gov/geo/query/acc.cgi?acc=GSE63789	25538139
Saccharomyces cerevisiae	GSE45366	https://www.ncbi.nlm.nih.gov/geo/query/acc.cgi?acc=GSE45366	23935536
Homo sapiens	GSE65885	https://www.ncbi.nlm.nih.gov/geo/query/acc.cgi?acc=GSE30881	26687005
Homo sapiens	GSE97140	https://www.ncbi.nlm.nih.gov/geo/query/acc.cgi?acc=GSE30919	28520920
Homo sapiens	GSE93133	https://www.ncbi.nlm.nih.gov/geo/query/acc.cgi?acc=GSE30916	28108655
Homo sapiens	GSE97384	https://www.ncbi.nlm.nih.gov/geo/query/acc.cgi?acc=GSE30920	28494858
Homo sapiens	GSE55195	https://www.ncbi.nlm.nih.gov/geo/query/acc.cgi?acc=GSE30864	25621764
Homo sapiens	GSE73136	https://www.ncbi.nlm.nih.gov/geo/query/acc.cgi?acc=GSE30895	26657557
Homo sapiens	GSE60426	https://www.ncbi.nlm.nih.gov/geo/query/acc.cgi?acc=GSE30873	25263593
Homo sapiens	GSE58207	https://www.ncbi.nlm.nih.gov/geo/query/acc.cgi?acc=GSE30867	25510491
Homo sapiens	GSE66927	https://www.ncbi.nlm.nih.gov/geo/query/acc.cgi?acc=GSE30884	26538417
Homo sapiens	GSE51584	https://www.ncbi.nlm.nih.gov/geo/query/acc.cgi?acc=GSE30860	25070500
Homo sapiens	GSE37744	https://www.ncbi.nlm.nih.gov/geo/query/acc.cgi?acc=GSE30846	22836135
Homo sapiens	GSE49339	https://www.ncbi.nlm.nih.gov/geo/query/acc.cgi?acc=GSE30857	23453015
Homo sapiens	SRA160745	https://www.ncbi.nlm.nih.gov/geo/query/acc.cgi?acc=GSE30926	25486063
Homo sapiens	GSE61375	https://www.ncbi.nlm.nih.gov/geo/query/acc.cgi?acc=GSE30875	25273840
Homo sapiens	GSE70211	https://www.ncbi.nlm.nih.gov/geo/query/acc.cgi?acc=GSE30891	27309803
Homo sapiens	GSE59821	https://www.ncbi.nlm.nih.gov/geo/query/acc.cgi?acc=GSE30869	26878238
Homo sapiens	GSE85864	https://www.ncbi.nlm.nih.gov/geo/query/acc.cgi?acc=GSE30913	27681415
Homo sapiens	GSE70802	https://www.ncbi.nlm.nih.gov/geo/query/acc.cgi?acc=GSE30892	27058758
Homo sapiens	GSE75290	https://www.ncbi.nlm.nih.gov/geo/query/acc.cgi?acc=GSE30903	27232982
Homo sapiens	GSE68008	https://www.ncbi.nlm.nih.gov/geo/query/acc.cgi?acc=GSE30888	26164698
Homo sapiens	GSE86214	https://www.ncbi.nlm.nih.gov/geo/query/acc.cgi?acc=GSE30914	28106072
Homo sapiens	GSE41605	https://www.ncbi.nlm.nih.gov/geo/query/acc.cgi?acc=GSE30847	23180859
Homo sapiens	GSE48785	https://www.ncbi.nlm.nih.gov/geo/query/acc.cgi?acc=GSE30855	24171104
Homo sapiens	GSE56887	https://www.ncbi.nlm.nih.gov/geo/query/acc.cgi?acc=GSE30865	25079319
Homo sapiens	GSE56924	https://www.ncbi.nlm.nih.gov/geo/query/acc.cgi?acc=GSE30866	26338483
Homo sapiens	GSE65778	https://www.ncbi.nlm.nih.gov/geo/query/acc.cgi?acc=GSE30880	25719440
Homo sapiens	GSE67902	https://www.ncbi.nlm.nih.gov/geo/query/acc.cgi?acc=GSE30887	26305499
Homo sapiens	GSE69906	https://www.ncbi.nlm.nih.gov/geo/query/acc.cgi?acc=GSE30890	26599541
Homo sapiens	GSE62247	https://www.ncbi.nlm.nih.gov/geo/query/acc.cgi?acc=GSE30876	26399832
Homo sapiens	GSE52976	https://www.ncbi.nlm.nih.gov/geo/query/acc.cgi?acc=GSE30862	25366541
Homo sapiens	GSE64962	https://www.ncbi.nlm.nih.gov/geo/query/acc.cgi?acc=GSE30879	26729373
Homo sapiens	GSE73565	https://www.ncbi.nlm.nih.gov/geo/query/acc.cgi?acc=GSE30897	26898226
Homo sapiens	GSE94460	https://www.ncbi.nlm.nih.gov/geo/query/acc.cgi?acc=GSE94460	29170441

Homo sapiens	GSE102113	https://www.ncbi.nlm.nih.gov/geo/query/acc.cgi?acc=GSE102113	30449621
Homo sapiens	GSE131809	https://www.ncbi.nlm.nih.gov/geo/query/acc.cgi?acc=GSE131809	31959994
Homo sapiens	GSE132725	https://www.ncbi.nlm.nih.gov/geo/query/acc.cgi?acc=GSE132725	31340047
Homo sapiens	GSE134752	https://www.ncbi.nlm.nih.gov/geo/query/acc.cgi?acc=GSE134752	
Homo sapiens	GSE22004	https://www.ncbi.nlm.nih.gov/geo/query/acc.cgi?acc=GSE22004	20703300
Homo sapiens	GSE39561	https://www.ncbi.nlm.nih.gov/geo/query/acc.cgi?acc=GSE39561	22879431
Homo sapiens	GSE48933	https://www.ncbi.nlm.nih.gov/geo/query/acc.cgi?acc=GSE48933	24301020
Homo sapiens	GSE51424	https://www.ncbi.nlm.nih.gov/geo/query/acc.cgi?acc=GSE51424	25122893
Homo sapiens	GSE52809	https://www.ncbi.nlm.nih.gov/geo/query/acc.cgi?acc=GSE52809	24476825
Homo sapiens	GSE61012	https://www.ncbi.nlm.nih.gov/geo/query/acc.cgi?acc=GSE61012	25378630
Homo sapiens	SRA056377	https://www.ncbi.nlm.nih.gov/sra/?term=SRA056377	22927429
Homo sapiens	SRA061778	https://trace.ddbj.nig.ac.jp/DRASearch/submission?acc=SRA061778	23290916
Homo sapiens	GSE103719	https://www.ncbi.nlm.nih.gov/geo/query/acc.cgi?acc=GSE103719	30257221
Homo sapiens	GSE133111	https://www.ncbi.nlm.nih.gov/geo/query/acc.cgi?acc=GSE133111	
Homo sapiens	GSE143623	https://www.ncbi.nlm.nih.gov/geo/query/acc.cgi?acc=GSE143623	32029688
Homo sapiens	GSE45833	https://www.ncbi.nlm.nih.gov/geo/query/acc.cgi?acc=GSE45833	23594524
Homo sapiens	GSE60040	https://www.ncbi.nlm.nih.gov/geo/query/acc.cgi?acc=GSE60040	25989971
Homo sapiens	GSE63570	https://www.ncbi.nlm.nih.gov/geo/query/acc.cgi?acc=GSE63570	25896322
Homo sapiens	GSE105082	https://www.ncbi.nlm.nih.gov/geo/query/acc.cgi?acc=GSE105082	30591072
Homo sapiens	GSE60095	https://www.ncbi.nlm.nih.gov/geo/query/acc.cgi?acc=GSE60095	25159147
Homo sapiens	GSE65912	https://www.ncbi.nlm.nih.gov/geo/query/acc.cgi?acc=GSE65912	26297486
Homo sapiens	GSE105172	https://www.ncbi.nlm.nih.gov/geo/query/acc.cgi?acc=GSE105172	31160600
Homo sapiens	GSE79664	https://www.ncbi.nlm.nih.gov/geo/query/acc.cgi?acc=GSE79664	27153541
Homo sapiens	GSE110323	https://www.ncbi.nlm.nih.gov/geo/query/acc.cgi?acc=GSE110323	30867593
Homo sapiens	GSE111866	https://www.ncbi.nlm.nih.gov/geo/query/acc.cgi?acc=GSE111866	30102689
Homo sapiens	GSE112276	https://www.ncbi.nlm.nih.gov/geo/query/acc.cgi?acc=GSE112276	31031084
Homo sapiens	GSE112353	https://www.ncbi.nlm.nih.gov/geo/query/acc.cgi?acc=GSE112353	30355487
Homo sapiens	GSE114636	https://www.ncbi.nlm.nih.gov/geo/query/acc.cgi?acc=GSE114636	30673779
Homo sapiens	GSE115146	https://www.ncbi.nlm.nih.gov/geo/query/acc.cgi?acc=GSE115146	30297778
Homo sapiens	GSE118050	https://www.ncbi.nlm.nih.gov/geo/query/acc.cgi?acc=GSE118050	30260431
Homo sapiens	GSE122461	https://www.ncbi.nlm.nih.gov/geo/query/acc.cgi?acc=GSE122461	30640896
Homo sapiens	GSE123564	https://www.ncbi.nlm.nih.gov/geo/query/acc.cgi?acc=GSE123564	30707697
Homo sapiens	GSE125218	https://www.ncbi.nlm.nih.gov/geo/query/acc.cgi?acc=GSE125218	31819274
Homo sapiens	GSE129869	https://www.ncbi.nlm.nih.gov/geo/query/acc.cgi?acc=GSE129869	31167946
Homo sapiens	GSE130781	https://www.ncbi.nlm.nih.gov/geo/query/acc.cgi?acc=GSE130781	
Homo sapiens	GSE131112	https://www.ncbi.nlm.nih.gov/geo/query/acc.cgi?acc=GSE131112	31284728
Danio rerio	GSE34743	https://www.ncbi.nlm.nih.gov/geo/query/acc.cgi?acc=GSE30844	22422859
Danio rerio	GSE46512	https://www.ncbi.nlm.nih.gov/geo/query/acc.cgi?acc=GSE30854	23698349
Danio rerio	GSE47558	https://www.ncbi.nlm.nih.gov/geo/query/acc.cgi?acc=GSE47558	24056933
Danio rerio	GSE52809	https://www.ncbi.nlm.nih.gov/geo/query/acc.cgi?acc=GSE52809	24476825
Danio rerio	GSE53693	https://www.ncbi.nlm.nih.gov/geo/query/acc.cgi?acc=GSE53693	24705786
Mus musculus	GSE83823	https://www.ncbi.nlm.nih.gov/geo/query/acc.cgi?acc=GSE83823	27899360
Mus musculus	GSE72066	https://www.ncbi.nlm.nih.gov/geo/query/acc.cgi?acc=GSE72066	27306184
Mus musculus	GSE50983	https://www.ncbi.nlm.nih.gov/geo/query/acc.cgi?acc=GSE50983	25063675

Mus musculus	GSE74139	https://www.ncbi.nlm.nih.gov/geo/query/acc.cgi?acc=GSE74139	26638175
Mus musculus	GSE84112	https://www.ncbi.nlm.nih.gov/geo/query/acc.cgi?acc=GSE84112	27956496
Mus musculus	SRA160745	https://trace.ddbj.nig.ac.jp/DRASearch/submission?acc=SRA160745	25486063
Mus musculus	GSE82220	https://www.ncbi.nlm.nih.gov/geo/query/acc.cgi?acc=GSE82220	27638886
Mus musculus	GSE51424	https://www.ncbi.nlm.nih.gov/geo/query/acc.cgi?acc=GSE51424	25122893
Mus musculus	GSE78163	https://www.ncbi.nlm.nih.gov/geo/query/acc.cgi?acc=GSE78163	27380875
Mus musculus	GSE30839	https://www.ncbi.nlm.nih.gov/geo/query/acc.cgi?acc=GSE30839	22056041
Mus musculus	GSE60095	https://www.ncbi.nlm.nih.gov/geo/query/acc.cgi?acc=GSE60095	25159147
Mus musculus	GSE74683	https://www.ncbi.nlm.nih.gov/geo/query/acc.cgi?acc=GSE74683	26651292
Mus musculus	SRA056377	https://www.ncbi.nlm.nih.gov/sra/?term=SRA056377	22927429
Mus musculus	GSE72851	https://www.ncbi.nlm.nih.gov/geo/query/acc.cgi?acc=GSE72851	28225755
Mus musculus	GSE53743	https://www.ncbi.nlm.nih.gov/geo/query/acc.cgi?acc=GSE53743	25215492
Mus musculus	GSE69800	https://www.ncbi.nlm.nih.gov/geo/query/acc.cgi?acc=GSE69800	27161320
Mus musculus	GSE89108	https://www.ncbi.nlm.nih.gov/geo/query/acc.cgi?acc=GSE89108	28720757
Mus musculus	GSE83332	https://www.ncbi.nlm.nih.gov/geo/query/acc.cgi?acc=GSE83332	28077873
Mus musculus	GSE36892	https://www.ncbi.nlm.nih.gov/geo/query/acc.cgi?acc=GSE36892	22552098
Mus musculus	GSE46038	https://www.ncbi.nlm.nih.gov/geo/query/acc.cgi?acc=GSE46038	23696641
Mus musculus	GSE41785	https://www.ncbi.nlm.nih.gov/geo/query/acc.cgi?acc=GSE41785	23766421
Mus musculus	GSE73369	https://www.ncbi.nlm.nih.gov/geo/query/acc.cgi?acc=GSE73369	26443847
Mus musculus	GSE103667	https://www.ncbi.nlm.nih.gov/geo/query/acc.cgi?acc=GSE103667	29576526
Mus musculus	GSE115106	https://www.ncbi.nlm.nih.gov/geo/query/acc.cgi?acc=GSE115106	30728504
Mus musculus	GSE105147	https://www.ncbi.nlm.nih.gov/geo/query/acc.cgi?acc=GSE105147	30643286
Mus musculus	GSE115110	https://www.ncbi.nlm.nih.gov/geo/query/acc.cgi?acc=GSE115110	31296853
Mus musculus	GSE106529	https://www.ncbi.nlm.nih.gov/geo/query/acc.cgi?acc=GSE106529	31186416
Mus musculus	GSE119365	https://www.ncbi.nlm.nih.gov/geo/query/acc.cgi?acc=GSE119365	31033440
Mus musculus	GSE124280	https://www.ncbi.nlm.nih.gov/geo/query/acc.cgi?acc=GSE124280	31924774
Mus musculus	GSE129816	https://www.ncbi.nlm.nih.gov/geo/query/acc.cgi?acc=GSE129816	31483294
Mus musculus	GSE129818	https://www.ncbi.nlm.nih.gov/geo/query/acc.cgi?acc=GSE129818	31483294
Mus musculus	GSE130898	https://www.ncbi.nlm.nih.gov/geo/query/acc.cgi?acc=GSE130898	31371437
Mus musculus	GSE141599	https://www.ncbi.nlm.nih.gov/geo/query/acc.cgi?acc=GSE141599	31999954
Mus musculus	GSE142802	https://www.ncbi.nlm.nih.gov/geo/query/acc.cgi?acc=GSE142802	
Mus musculus	GSE52809	https://www.ncbi.nlm.nih.gov/geo/query/acc.cgi?acc=GSE52809	24476825
Mus musculus	GSE58423	https://www.ncbi.nlm.nih.gov/geo/query/acc.cgi?acc=GSE58423	25380226
Mus musculus	GSE59793	https://www.ncbi.nlm.nih.gov/geo/query/acc.cgi?acc=GSE59793	25745177
Mus musculus	GSE67305	https://www.ncbi.nlm.nih.gov/geo/query/acc.cgi?acc=GSE67305	26486724
Mus musculus	PRJEB7207	http://www.ebi.ac.uk/ena/data/view/PRJEB7207	25873627
Mus musculus	GSE22004	https://www.ncbi.nlm.nih.gov/geo/query/acc.cgi?acc=GSE22004	20703300
Rattus norvegicus	GSE55195	https://www.ncbi.nlm.nih.gov/geo/query/acc.cgi?acc=GSE55195	25621764
Rattus norvegicus	GSE66715	https://www.ncbi.nlm.nih.gov/geo/query/acc.cgi?acc=GSE66715	27135913
Rattus norvegicus	GSE110426	https://www.ncbi.nlm.nih.gov/geo/query/acc.cgi?acc=GSE110426	29878763
Drosophila melanogaster	GSE60384	https://www.ncbi.nlm.nih.gov/geo/query/acc.cgi?acc=GSE30872	25144939
Drosophila melanogaster	GSE49197	https://www.ncbi.nlm.nih.gov/geo/query/acc.cgi?acc=GSE30856	24302569
Drosophila melanogaster	GSE52799	https://www.ncbi.nlm.nih.gov/geo/query/acc.cgi?acc=GSE52799	24882012

Caenorhabditis elegans	GSE62859	https://www.ncbi.nlm.nih.gov/geo/query/acc.cgi?acc=GSE30877	25378320
Caenorhabditis elegans	GSE52910	https://www.ncbi.nlm.nih.gov/geo/query/acc.cgi?acc=GSE30861	24440504
Caenorhabditis elegans	GSE67387	https://www.ncbi.nlm.nih.gov/geo/query/acc.cgi?acc=GSE30885	26052047
Caenorhabditis elegans	SRA049309	https://www.ncbi.nlm.nih.gov/geo/query/acc.cgi?acc=GSE30921	22045228
Caenorhabditis elegans	SRA055804	https://www.ncbi.nlm.nih.gov/geo/query/acc.cgi?acc=GSE30923	22855835

Back to the table of contents

Supplementary S5

Getting started tutorials for OpenProt v1.6

The tutorials below give detailed instructions to navigate the genome browser, the query interface, the submission platform and the downloads interface on OpenProt v1.6.

*** Getting started with the **genome browser** in OpenProt ***

If you want to browse the genome of a specific species for AltORFs (all predicted and those with evidence of expression), click **Browse**.

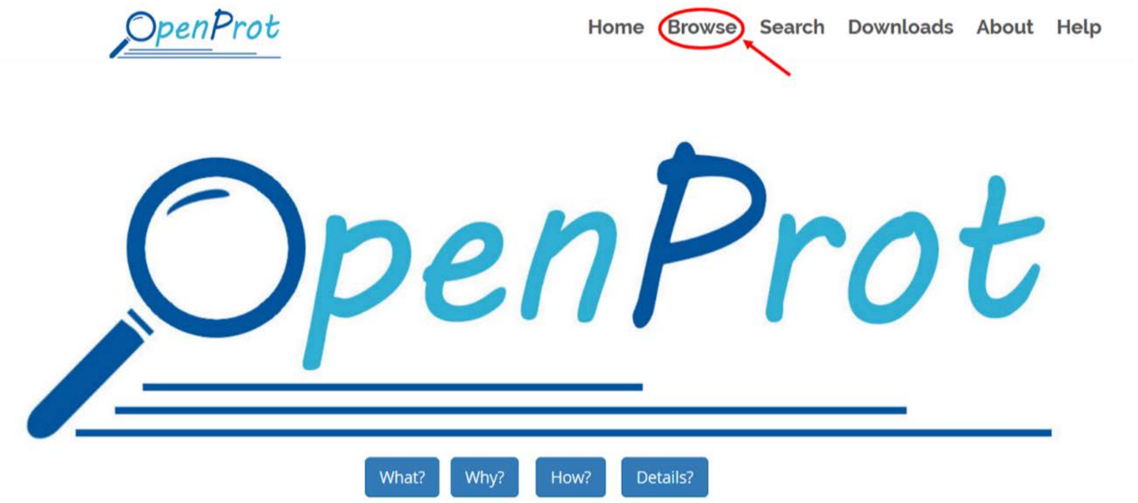

You will then be directed towards a query page.

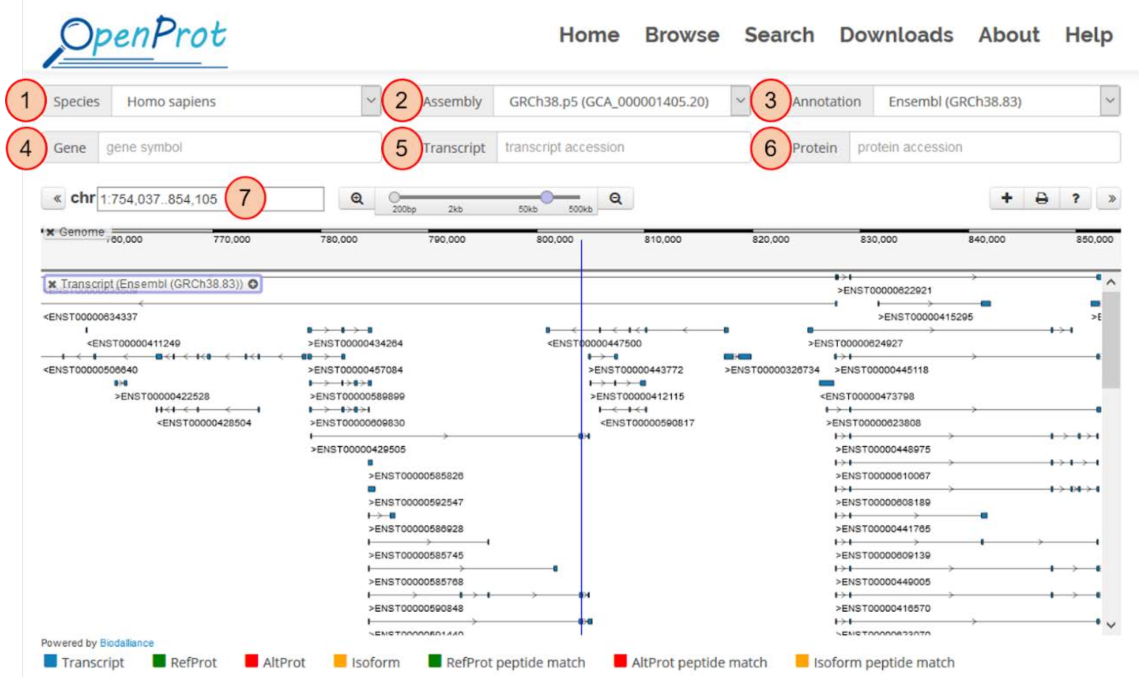

You can input your search criteria as follow:

1. Select a **species** *(default is Homo sapiens)*.
2. Select an **assembly** *(default is the most recent in each species)*.
3. Select an **annotation** *(default is Ensembl)*. Both Ensembl and NCBI RefSeq annotations are used by OpenProt to predict AltProts, and the browser is available for both. *If you want to know why OpenProt supports multiple annotations, you can click [here](). For more informations on how to display both annotations on the browser, click [here]().*
4. Enter the name of your **gene** of interest.

Alternatively, you can also search by **transcript** or **protein** accessions (**5** and **6** respectively). Both Ensembl and RefSeq accession IDs are accepted (depending on the chosen annotation). You can also directly enter genomic coordinates of interest (**7**).

Below is an example for the *COL1A1* gene. Once you have entered your gene name (**1**) and launched the search (**2**), your results will appear centered in the browser window.

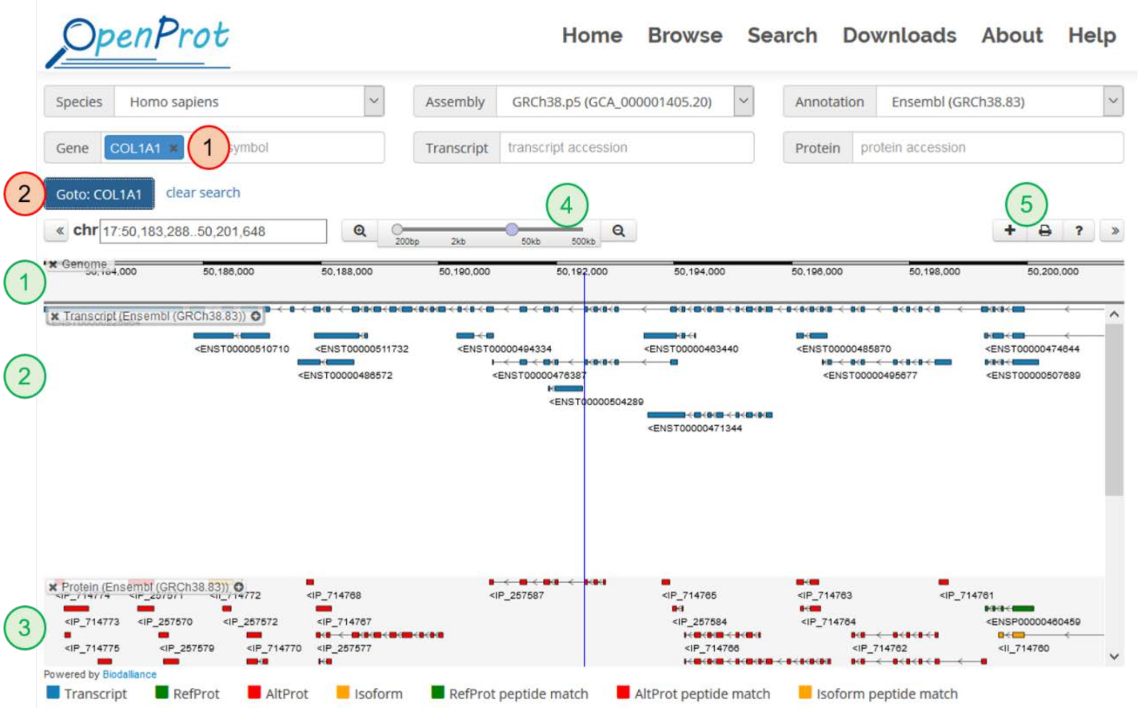

You can visualize the genomic coordinates (**1**) and the different tracks. The first track contains transcripts for the chosen annotations (**2** - here, Ensembl). The second contains predicted proteins (**3**). The colour code is indicated below the browser with the transcripts annotated in blue, the RefProt in green, the AltProt in red and the Novel Isoforms in yellow. You can widen or narrow the browser window (**4**) and custom your display by adding or removing a track from the registry (**5**) The registry includes: genome, transcript, protein and peptide detection *by default*.

If you scroll down on the genome browser (**1**), the last track will appear and contains the peptide detected by MS (**1**).

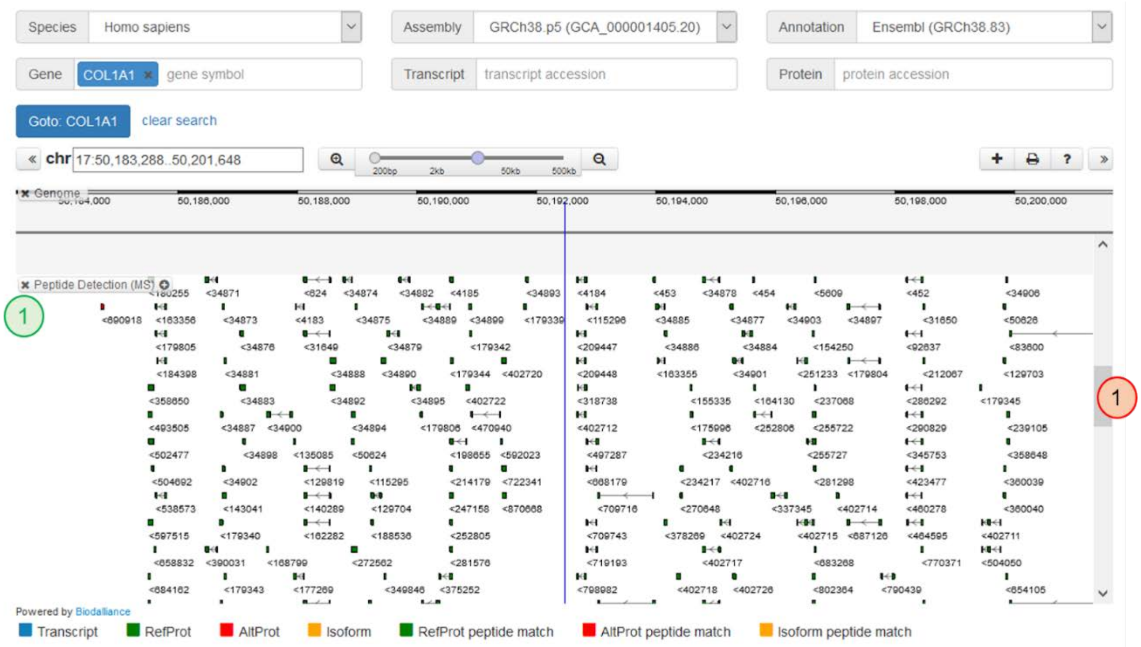

Furthermore, you can **click on a peptide** and this will display the details associated to this peptide in a pop-up window.

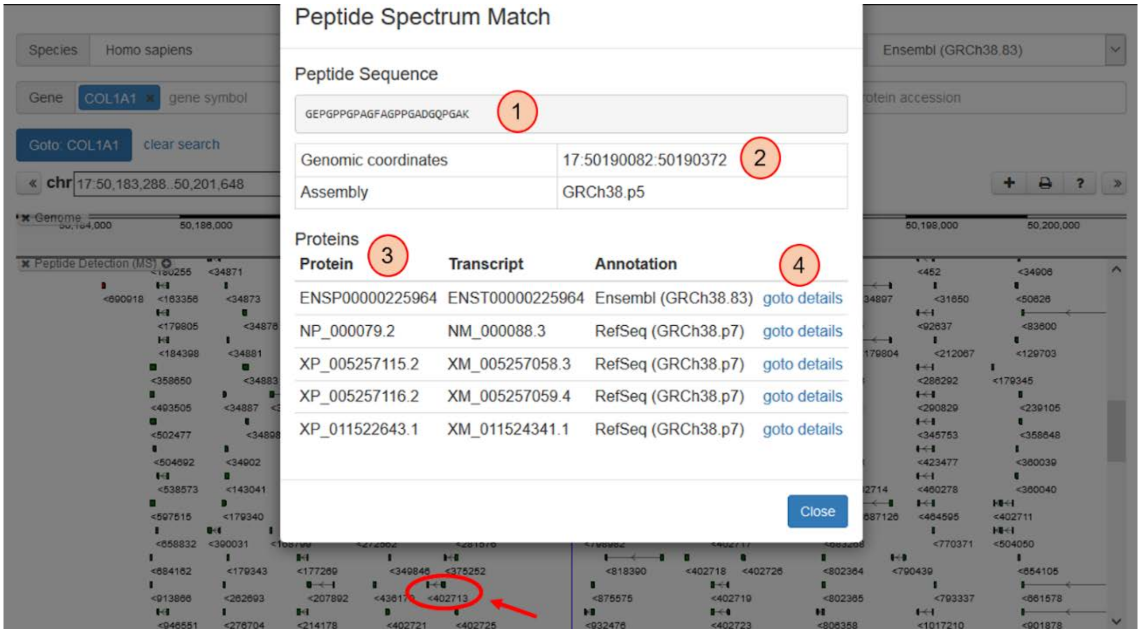

The pop-up windows displays the peptide sequence (**1**), its genomic coordinates (**2**) and the proteins assigned to that peptide (**3**). All proteins this peptide has been assigned to are listed, across both annotations (Ensembl + RefSeq). *For more information on peptide assignation rules, click here*. The details page of the assigned proteins can be consulted directly by clicking on the goto details link (**4**).

Such pop-up windows are also displayed when clicking on a protein or a **transcript** (as shown below).

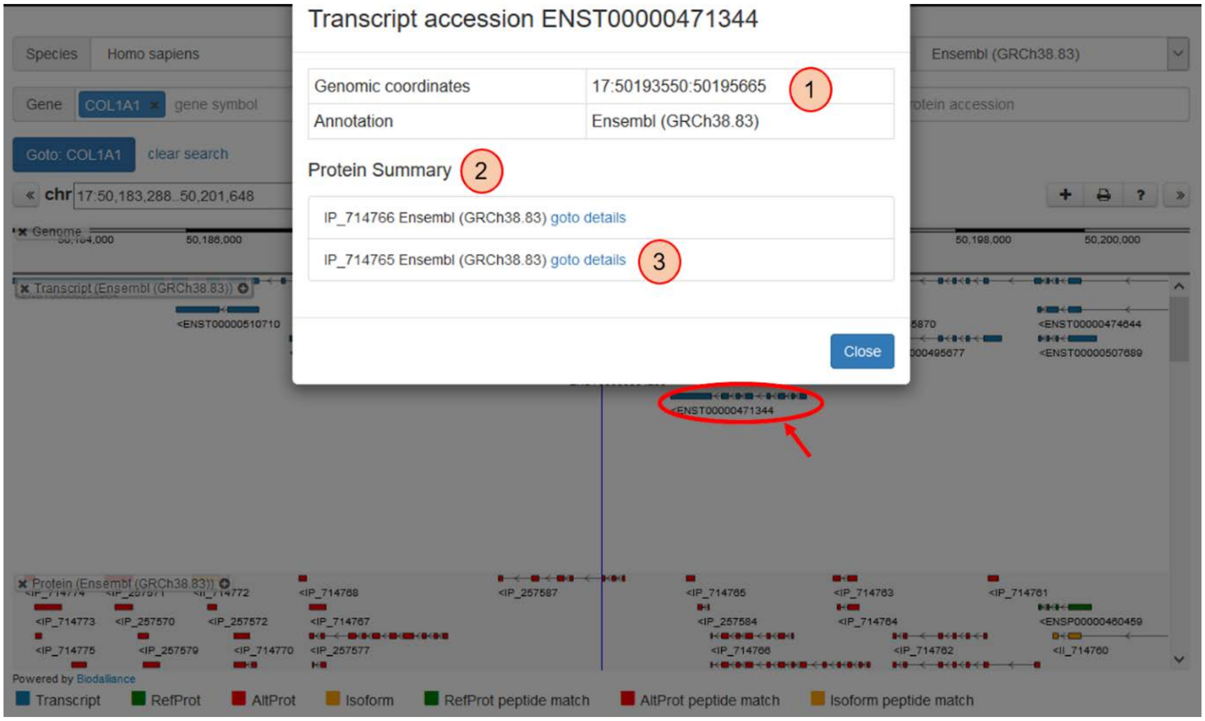

The transcript associated pop-up window contains the transcript genomic coordinates (**1**) and a list of all the associated proteins to this transcript (**2**). Each protein can then be accessed by clicking on the goto details link (**3**).

*** Getting started with the **query interface** in OpenProt ***

If you want to know if a specific gene contains AltORFs (all predicted and those with evidence of expression), click **Search**.

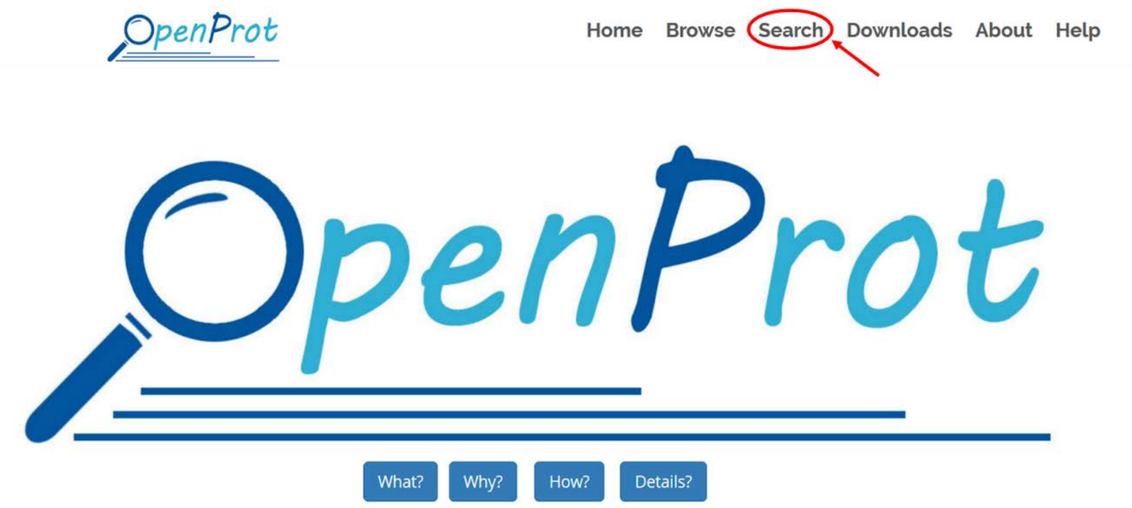

You will then be redirected towards a query page:

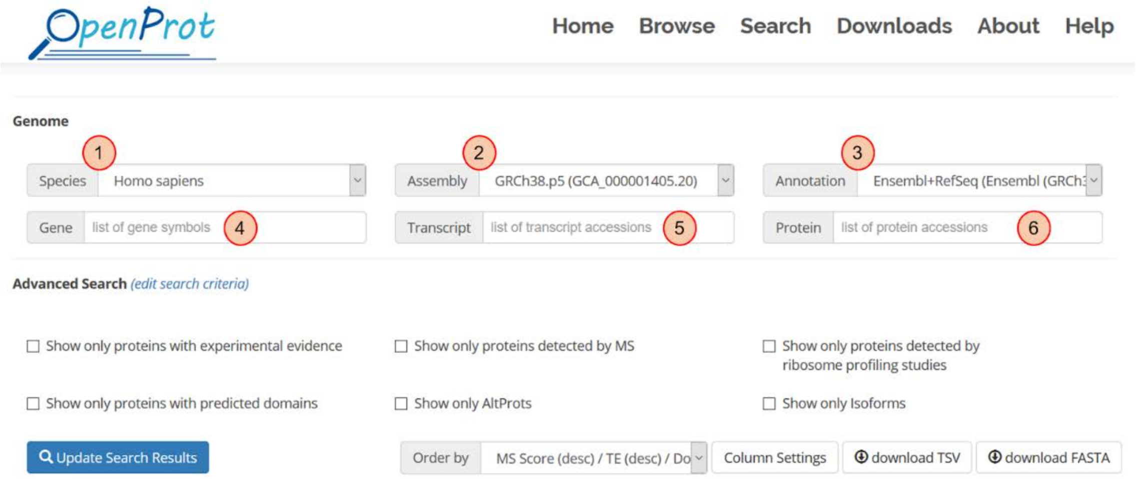

You can input your search criteria as follow:

1. Select a **species** *(default is Homo sapiens)*.
2. Select an **assembly** *(default is the most recent in each species)*.
3. Select an **annotation** *(default is Ensembl+RefSeq)*. Several annotations are used by OpenProt to predict AltProts. Ensembl, NCBI RefSeq and combined Ensembl+RefSeq annotations are available for all species. If you want to know why OpenProt supports multiple annotations, you can click here.
4. Enter the name of your **gene** of interest.

Alternatively, you can also search by **transcript** or **protein** accessions (**5** and **6** respectively). Both Ensembl and RefSeq accession IDs are accepted. Proteins may be searched on one or more specific transcripts.Similarly, one or more proteins can be searched for simultaneously.

Below is an example for the *COL1A1* gene. Once you have entered your gene name and launched the search, your results will appear below. The number of found proteins respecting your search criteria is indicated at the top (**1**) of your results table (**2**).

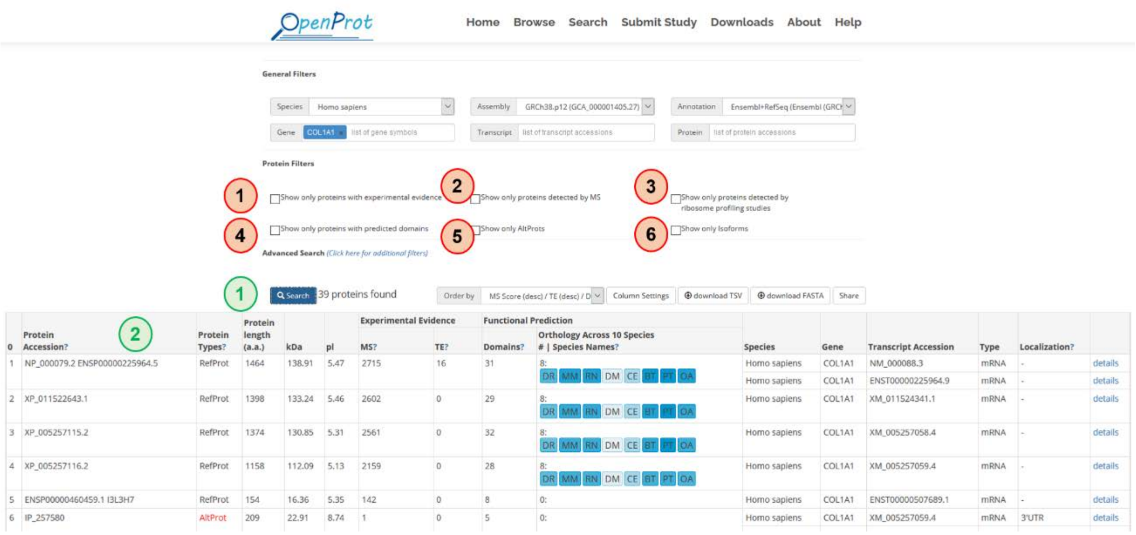

You can then refine your search results by playing with the options in the dropdown menu or by selecting the Advanced Search option.

1. Tick to search for or display only proteins (RefProts, AltProts and Isoforms) that **have been detected** by mass spectrometry (MS) and/or for which translation events (TE) have been identified in ribosome profiling studies.
2. Tick to search for or display only proteins that have been **detected by MS**. For a list of MS studies reanalysed by OpenProt, click here.
3. Tick to search for or display only proteins that have been **detected by ribosome profiling**. For a list of ribosome profiling studies reanalysed by OpenProt, click here.
4. Tick to search for or display only proteins **with predicted domains** by InterProScan.
5. Tick to search for or display **only AltProts**.
6. Tick to search for or display **only Isoforms**.

Any of the above can be combined as you wish. An advanced search is also available by clicking **Advanced Search**.

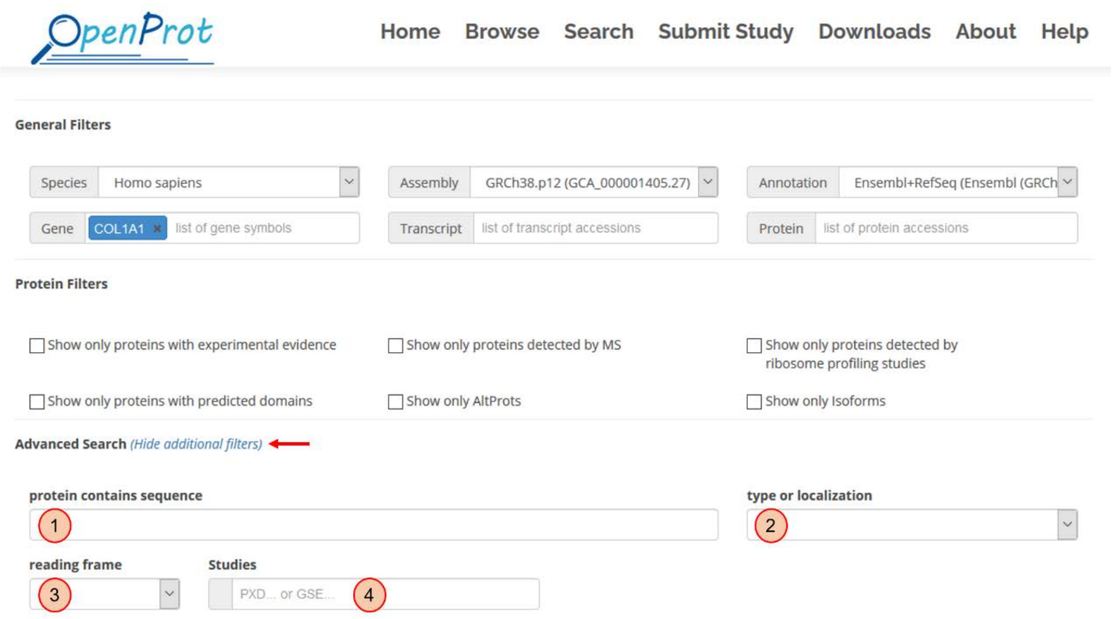

1. Filter by a specific **amino acid sequence**.
2. Filter according to the **transcript type** (mRNA or ncRNA) or the **localization** of AltORFs in transcripts. Within mRNAs, the localization of AltORFs is defined by the predicted start codon localization with respect to the annotated CDS start codon. The localization of AltORFs within non-coding RNAs is labeled "-". There are three possible choice of localizations of AltORFs within mRNAs: "*5'UTR*", "*CDS*", "*3'UTR*". Thus, the dropdown menu offers 5 choice: "*5'UTR*", "*3'UTR*", "*CDS*", "*ncRNA*", or "*mRNA*".
3. Filter AltORFs in a specific **reading frame** (+1, +2 or +3). The reading frame is determined with respect to the first nucleotide of each transcript (+1 reading frame).
4. Filter by dataset identifier. This is a dropdown menu containing all datasets currently in OpenProt. Select one to mine proteins detected in this dataset. *Please note that the filter supports only one study at a time.*

You can further sort your results by clicking on any option of the **Order by** dropdown menu (**1**).

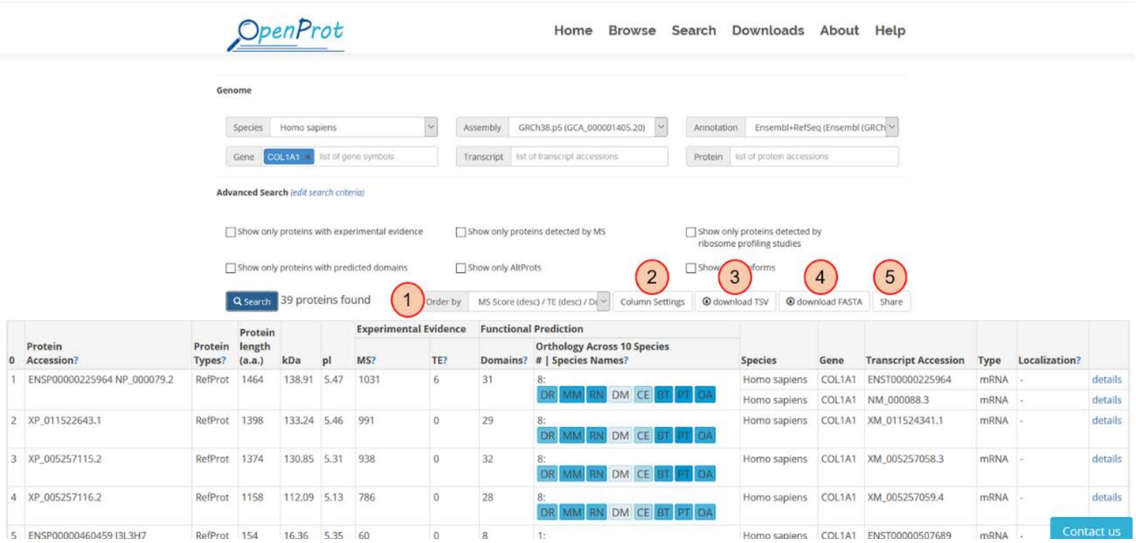

1. The following **sorting options** are available:
   “MS score (desc) / TE (desc) / Domains (desc)” *(by default)*;
   “Domains (desc) / MS score (desc) / TE (desc)”;
   “TE (desc) / MS score (desc) / Domains (desc)”;
   “Molecular Weight (asc) / MS score (desc) / TE (desc) / Domains (desc)”;
   “Molecular Weight (desc) / MS score (desc) / TE (desc) / Domains (desc)”;
   “Protein Length (asc) / MS score (desc) / TE (desc) / Domains (desc)”;
   “Protein Length (desc) / MS score (desc) / TE (desc) / Domains (desc)”.
2. Control which columns you want to see in the results table by clicking on the **Column Settings** and deselect any you don’t want to see.
3. You can download your results table by clicking on **Download as TSV**. *For more options and information on available downloads, click on Downloads Guidelines.*
4. You can download protein sequences from your results table by clicking on **Download as FASTA**. *For more options and information on available downloads, click on Downloads Guidelines.*

You can also share your search by clicking on **Share**. A pop-up window will display a shareable link.

*** Getting started with the **data submission platform** in OpenProt ***

From any OpenProt page, including the home page, click **Submit study**.

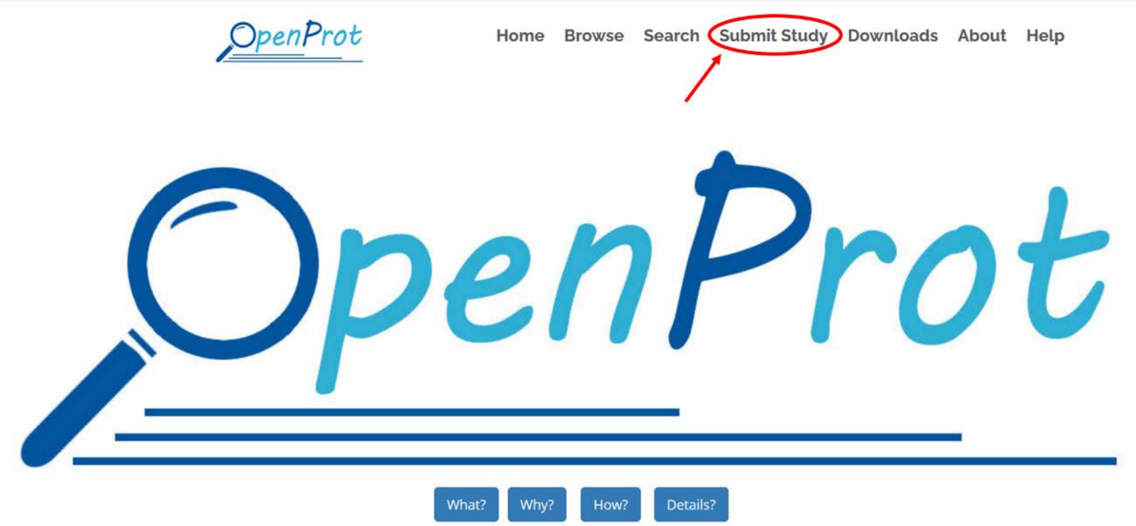

Once you clicked on Submit study, you should first select the type of file you are submitting: **mass spectrometry** or **ribosome profiling**.

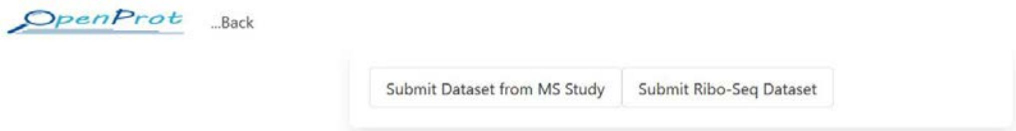

For **<u>mass spectrometry studies</u>**, your dataset has to be available in the PRIDE Archive with a public PXD accession.

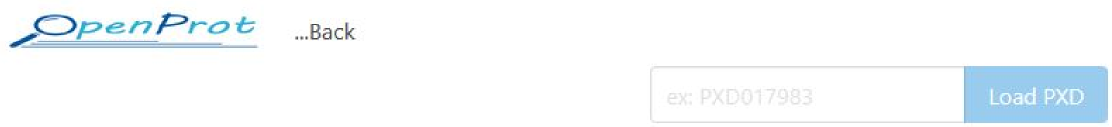

After entering the PXD accession number, the OpenProt submission platform will retrieve information from the PRIDE repository (here, we use the PXD015644 as an example). Thus, the **PMID** (**1**) and **citation** (**2**) are automatically filled, as well as the available **samples** in the dataset (**3**).

First, enter a **contact email** that will serve for all future correspondence (**1**). For example, we will send you the results of the analysis at this email address.

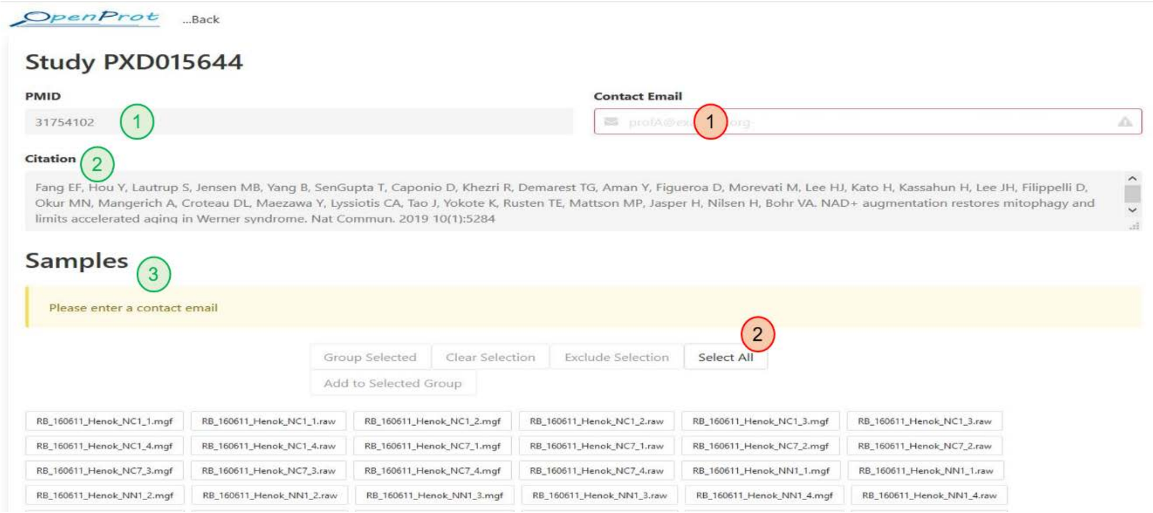

Once you have entered your email address, you can start **selecting samples** (**2**).

In order to select a sample, click on its name. The blue color indicates the sample is selected (**1**), the white background indicates the sample is not selected (**2**).

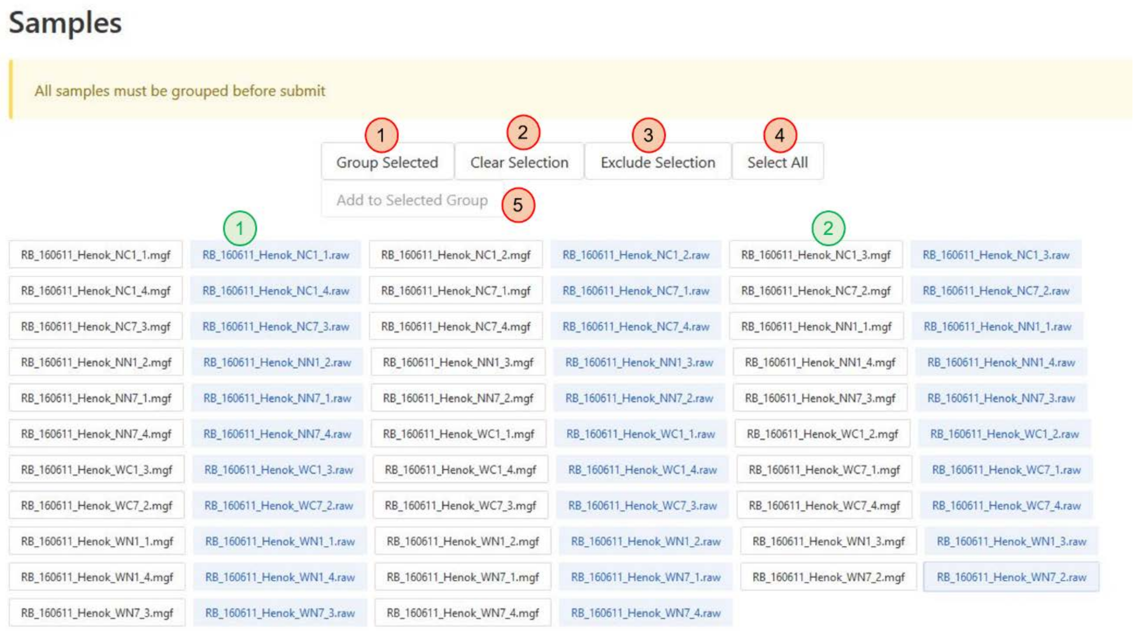

Once samples are selected, you can click on "**Group selected**" (**1**) to add each of them to on group with identical parameters. To correct erroneous selection, you can click on "**clear selection**" (**2**). If the datasets contain some samples that you don't want to include in the analysis, you can click on "**Exclude selection**" (**3**). If you want to select all samples at once, you can click on "**select all**" (**4**). Once samples are grouped, they will be removed from the selection panel. If you forgot one sample, you can add it to a pre-formed group by clicking on "**add to selected group**" (**5**). *Please not that all samples must be grouped in order to submit*.

Your grouped samples will then appear in the parameters editing box (**1**). Please note that your samples should be grouped by parameter settings.

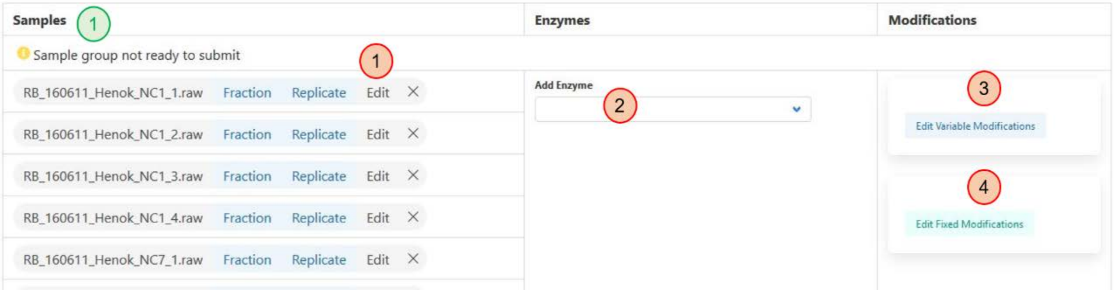

For each sample within a group, you can edit its fraction and replicate number by clicking on "**Edit**" (**1**). Then, for each group you have to indicate the **enzyme** (**2**) used for protein digestion, and the **variable** (**3**) and **fixed modifications** (**4**) to include in the analysis. These are drop-down menus with all available enzyme and modification in our pipeline. For custom enzyme or modifications, please contact us.

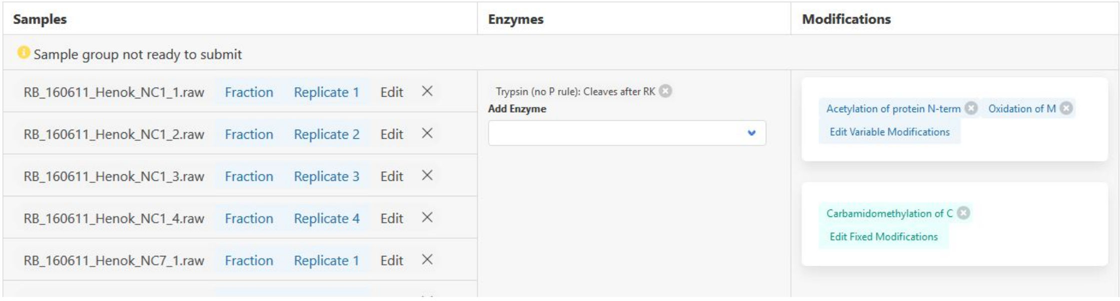

The parameters entered can always be changed by clicking the cross next to the selected enzyme or modification.

At the bottom of the page, the next parameters to enter are the **species** (**1**), the **type of biological sample** (**2**), the **fragmentation** protocol (**3**) and the **mass spectrometer** used (**4**).

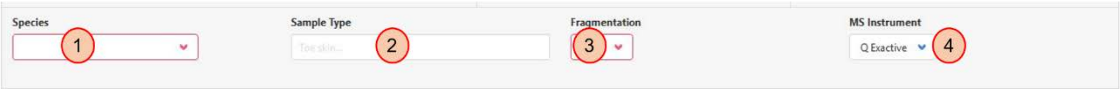

The species, fragmentation and MS instrument are compulsory for submission. The MS instrument is retrieved from the PRIDE directory. The species is a dropdown menu containing all species currently supported by OpenProt. The fragmentation protocol is a dropdown menu with the protocols currently supported by OpenProt. *(For more information on the fragmentation protocol, click here)*.

Once all compulsory parameters have been filled, you can click on submit. You will received an email (at the email address indicated at the top of the form) to confirm the submission (please check your spam folder if you don't receive any email).

For **ribosome profiling studies**, your dataset has to be available in the Gene Omnibus Archive with a public GSE accession.

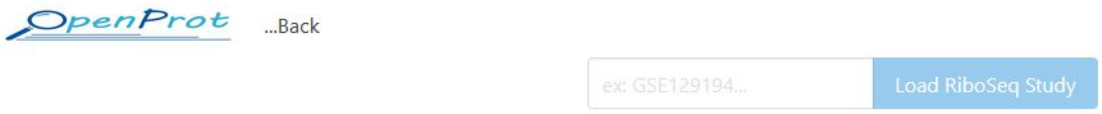

After entering the GSE accession number, the OpenProt submission platform will retrieve information from the Gene Omnibus repository (here, we use the GSE144682 as an example). Thus, the **PMID** (**1**) and **citation** (**2**) are automatically filled, as well as the available **samples** in the dataset (**3**).

First, enter a **contact email** that will serve for all future correspondence (**1**). For example, we will send you the results of the analysis at this email address.

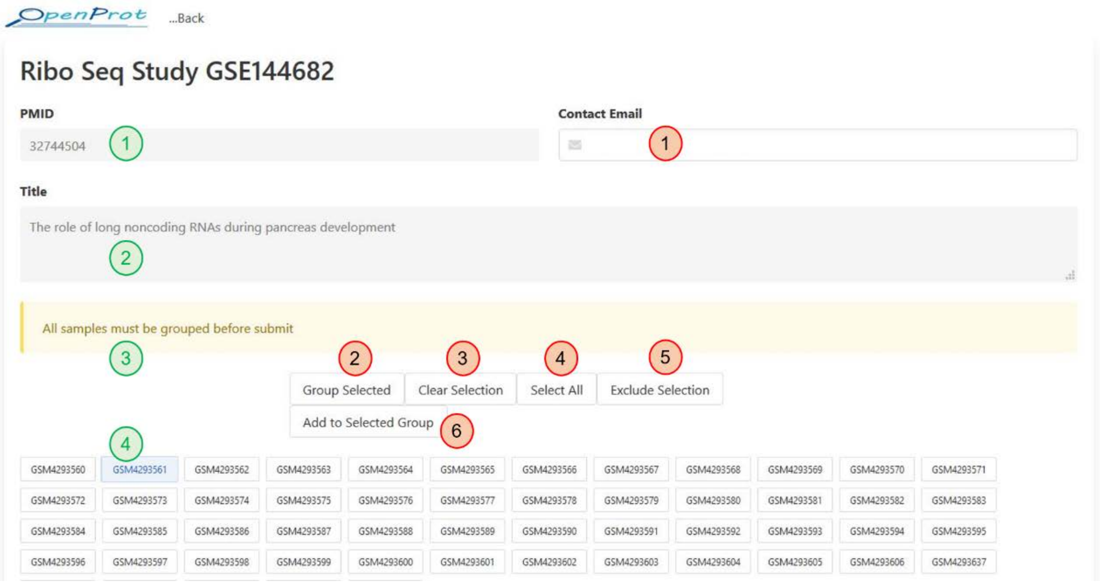

Once you have entered your email address, you can start **selecting samples**.

In order to select a sample, click on its name. The blue color indicates the sample is selected (**4**), the white background indicates the sample is not selected.

Once samples are selected, you can click on "**Group selected**" (**2**) to add each of them to on group with identical parameters. To correct erroneous selection, you can click on "**clear selection**" (**3**). If you want to select all samples at once, you can click on "**select all**" (**4**). If the datasets contain some samples that you don't want to include in the analysis, you can click on "**Exclude selection**" (**5**). Once samples are grouped, they will be removed from the selection panel. If you forgot one sample, you can add it to a pre-formed group by clicking on "**add to selected group**" (**6**). *Please not that all samples must be grouped in order to submit*.

Your grouped samples will then appear in the parameters editing box (**1**). Please note that your samples should be grouped by parameter settings.

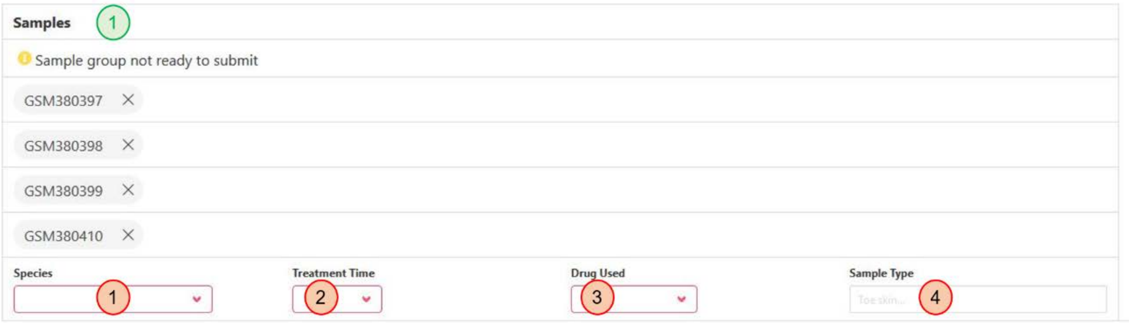

A sample can always be removed from the group by clicking the cross next to its name.

At the bottom of the page, the next parameters to enter are the **species** (**1**), the **time of treatment** (**2**), the **drug** used (**3**) and the **biological type of the sample** (**4**).

The species, time of treatment and drug used are compulsory for submission. The species is a dropdown menu containing all species currently supported by OpenProt. The time of treatment should correspond to when the drug was added during the protocol (if the drug was part of the lysis buffer, select n/a). The drug used is a dropdown menu containing all drugs currently supported by OpenProt.

Once all compulsory parameters have been filled, you can click on submit. You will received an email (at the email address indicated at the top of the form) to confirm the submission (please check your spam folder if you don't receive any email).

*** Getting started with the **downloads platform** in OpenProt ***

From any OpenProt page, including the home page, click **Downloads**.

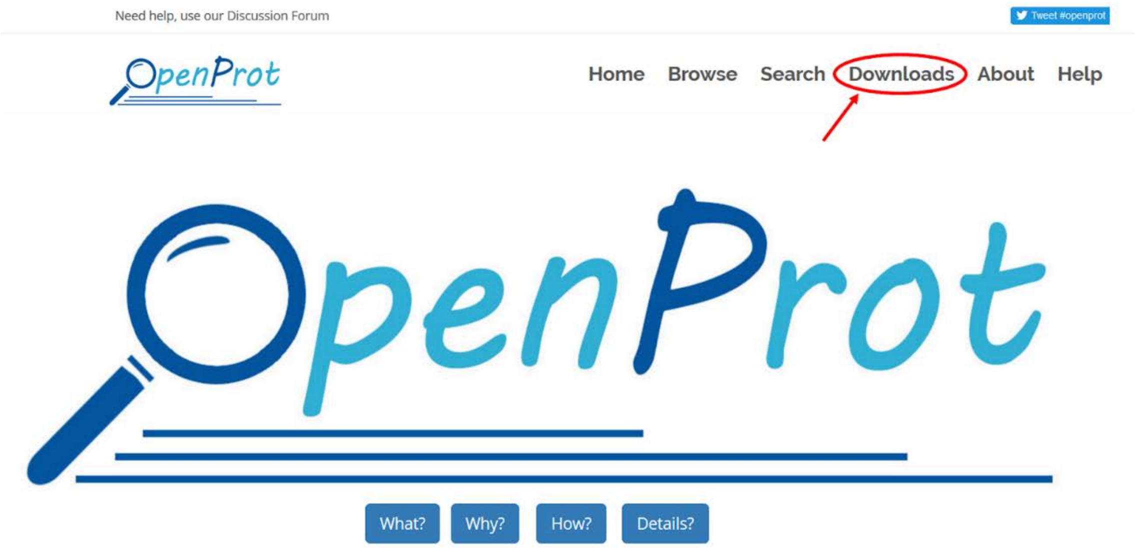

Once you clicked on Downloads, you should first select an **OpenProt release**. The most recent is the default option.

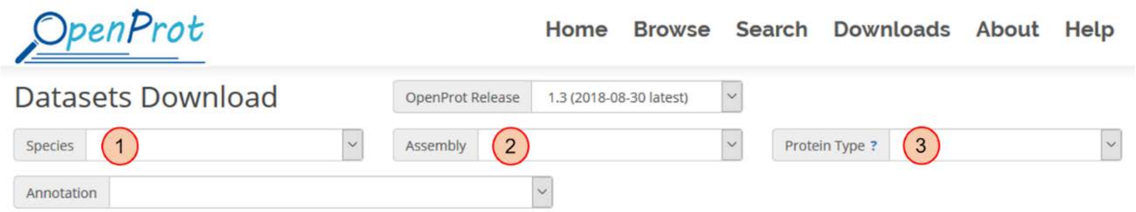

You can then tune the database you would like to download:

1. Select a **species**. Available species so far are: *Homo sapiens*, *Mus musculus*, *Rattus norvegicus*, *Pan troglodytes*, *Danio rerio*, *Drosophila melanogaster*, *Caenorhabditis elegans*, *Bos taurus*, *Saccharomyces cerevisiae S288c*, and *Ovis aries*.
2. Select an **assembly**. The most recent for each species is input by default upon species selection.
3. Select the desired **protein type**. You can choose whether you would like to download RefProts only, or AltProts and Isoforms only, or if you would like to download all, RefProts, AltProts and Isoforms.

Once you have selected the protein type you desire, a result table will already appear, but you can refine it further.

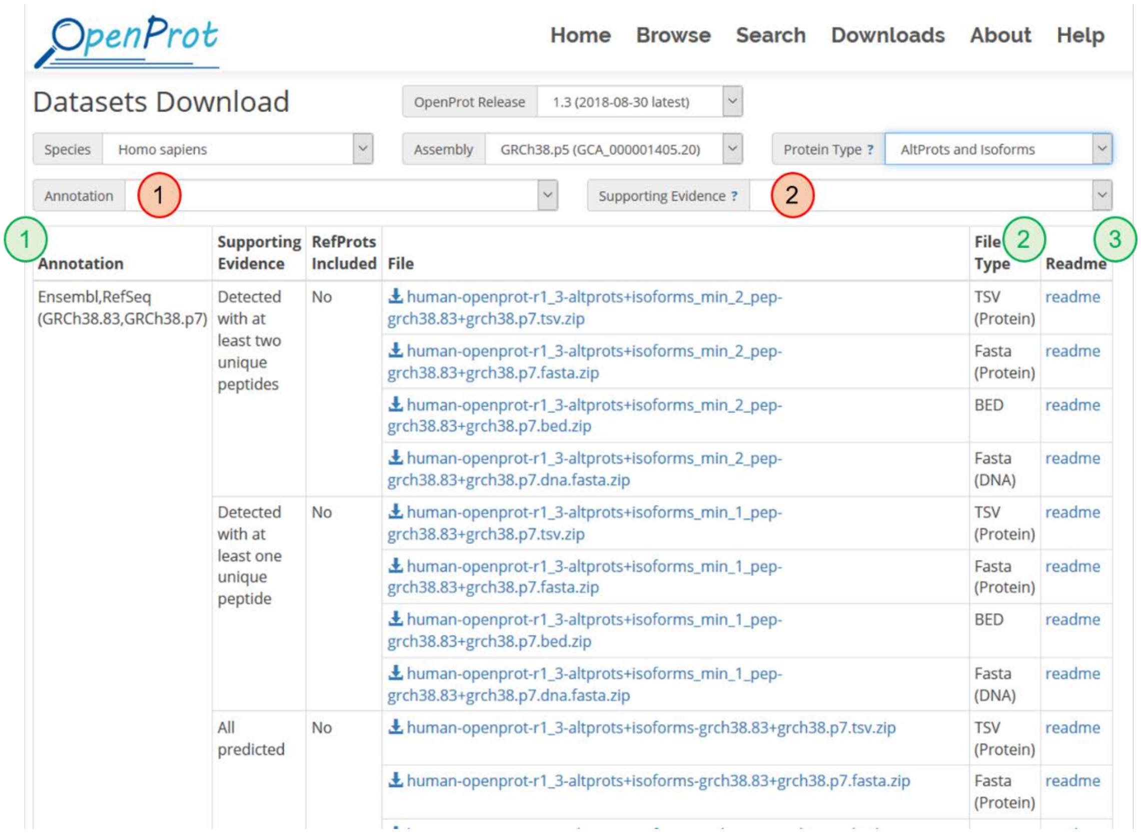

1. Select an **annotation**. OpenProt supports both Ensembl and NCBI RefSeq annotations for all species. If you would like to have more information on supported annotations, please click here.
2. Select the desired level of **supporting evidence**. You will be given this choice if you chose as protein type one that includes AltProts and Isoforms. You have 3 options: "*all predicted*", "*detected with at least one unique peptide*", or "*detected with at least two unique peptides*". This choice refers to the level of supporting MS evidence annotated in OpenProt database. If you are unsure which database would suit you best, you can read more here for recommendations on which one to choose.

The table of results from your download query are grouped on a table. The first column (**1**) indicates the annotation used, and the next two refers to your search criteria regarding supporting evidence and the protein type. Several file types are available for download (**2** - TSV, FASTA (protein), FASTA (DNA), or BED). Finally, each file is accompanied by a readme that regroups all information needed to understand it (**3**): headers, parse rules and file naming scheme.

Once you have selected the file you wish to download, a pop-up table containing the **downloadable file** becomes visible. You can **download** the database by clicking on its name, or from the read me pop-up.

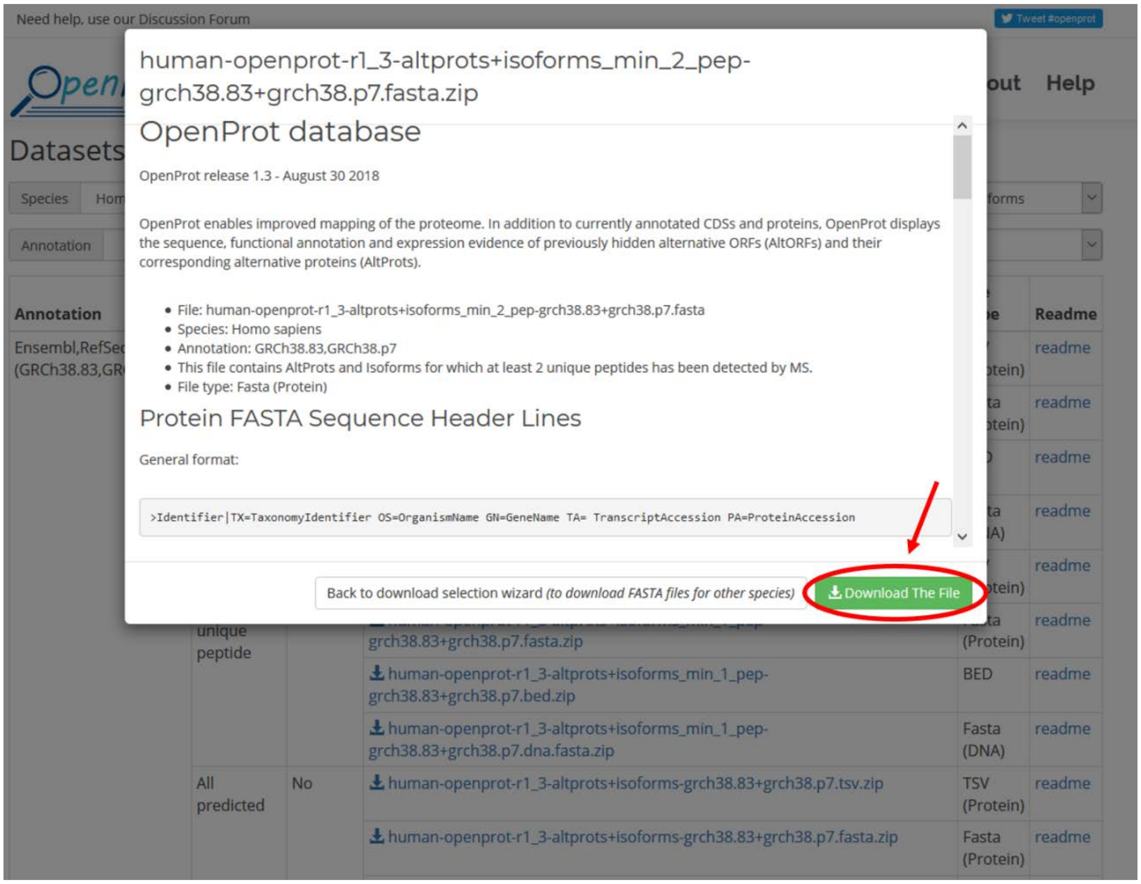

[Back to the table of contents]
